# The Impact of Metal Centers in the M-MOF-74 Series on Formic Acid Production

**DOI:** 10.1021/acsami.4c10678

**Published:** 2024-08-14

**Authors:** Dominika O. Wasik, José Manuel Vicent-Luna, Shima Rezaie, Azahara Luna-Triguero, Thijs J. H. Vlugt, Sofía Calero

**Affiliations:** † Materials Simulation and Modelling, Department of Applied Physics and Science Education, Eindhoven University of Technology, 5600MB Eindhoven, The Netherlands; ‡ Eindhoven Institute for Renewable Energy Systems, Eindhoven University of Technology, PO Box 513, 5600 MB Eindhoven, The Netherlands; § Energy Technology, Department of Mechanical Engineering, Eindhoven University of Technology, 5600MB Eindhoven, The Netherlands; ∥ Engineering Thermodynamics, Process & Energy Department, Faculty of Mechanical, Maritime and Materials Engineering, Delft University of Technology, Leeghwaterstraat 39, 2628CB Delft, The Netherlands

**Keywords:** molecular simulations, adsorption, hydrogenation, thermodynamic equilibrium, metal−organic frameworks

## Abstract

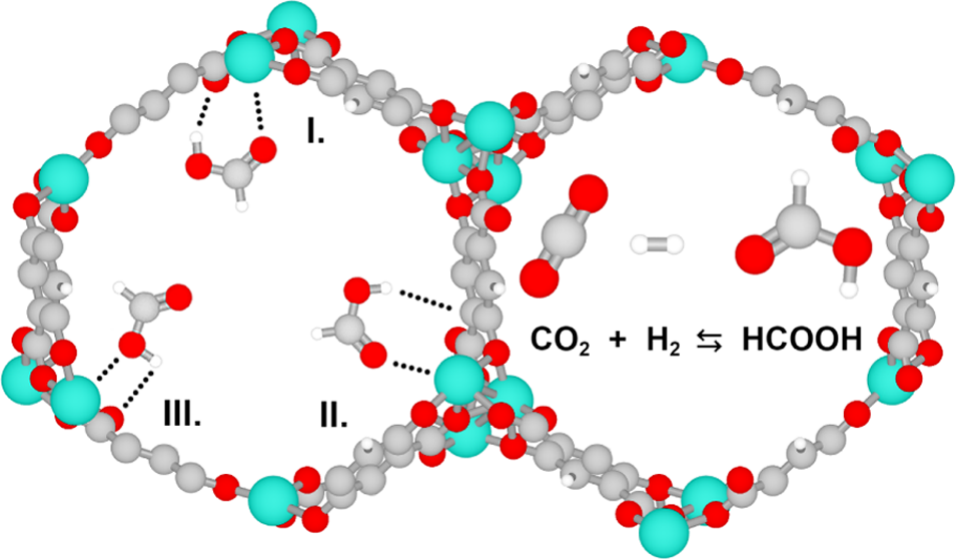

The confinement effect of porous materials on the thermodynamical equilibrium of the CO_2_ hydrogenation reaction presents a cost-effective alternative to transition metal catalysts. In metal–organic frameworks, the type of metal center has a greater impact on the enhancement of formic acid production than the scale of confinement resulting from the pore size. The M-MOF-74 series enables a comprehensive study of how different metal centers affect HCOOH production, minimizing the effect of pore size. In this work, molecular simulations were used to analyze the adsorption of HCOOH and the CO_2_ hydrogenation reaction in M-MOF-74, where M = Ni, Cu, Co, Fe, Mn, Zn. We combine classical simulations and density functional theory calculations to gain insights into the mechanisms that govern the low coverage adsorption of HCOOH in the surrounding of the metal centers of M-MOF-74. The impact of metal centers on the HCOOH yield was assessed by Monte Carlo simulations in the grand-canonical ensemble, using gas-phase compositions of CO_2_, H_2_, and HCOOH at chemical equilibrium at 298.15–800 K, 1–60 bar. The performance of M-MOF-74 in HCOOH production follows the same order as the uptake and the heat of HCOOH adsorption: Ni > Co > Fe > Mn > Zn > Cu. Ni-MOF-74 increases the mole fraction of HCOOH by ca. 10^5^ times compared to the gas phase at 298.15 K, 60 bar. Ni-MOF-74 has the potential to be more economically attractive for CO_2_ conversion than transition metal catalysts, achieving HCOOH production at concentrations comparable to the highest formate levels reported for transition metal catalysts and offering a more valuable molecular form of the product.

## 1 Introduction

In 2023, global energy-related CO_2_ emissions increased by 1.1%, reaching a new record of 37.4 billion tonnes (Gt).^1^ From 2019 to 2023, total energy-related CO_2_ emissions raised by ca. 900 million tonnes (Mt), however without the adoption of clean energy technologies these emissions would have grown 3-fold.^1^ Further advancement of sustainable energy technologies that significantly slow down emissions is an important topic for future research. An economically attractive method to reduce CO_2_ emissions is the capture at the source of production and the conversion to valuable chemicals, e.g., formic acid, salicylic acid, methanol, urea, propylene, and polyols.^2^ Due to the wide range of formic acid (HCOOH) applications, e.g., as a hydrogen carrier,^3^ fungicidal and bactericidal agent,^4^ in the production of rubber^5^ and the water–gas-shift reaction,^6^ the HCOOH global market value is forecast to increase from 1.8 billion dollars in 2023 to 2.8 billion dollars by 2033.^7^ One of the possible methods for HCOOH production that gained attention over the past few decades is the CO_2_ hydrogenation reaction:^8^ CO_2_ + H_2_ ⇄ HCOOH. The high free energy barrier of 351.46 kJ mol^–1^ for CO_2_ hydrogenation in the gas phase^9^ indicates that a catalyst is needed. The most efficient transition metal-based catalytic systems for CO_2_ hydrogenation involve catalysts with pincer ligands,^10,11^ half-sandwich catalysts with or without proton-responsive ligands,^12,13^ N-heterocyclic carbene ligands,^14,15^ and phosphine ligands.^16,17^ Given the significant limitations of transition metal catalysts, such as their cost and toxicity,^9^ it is essential to investigate methods to improve CO_2_ hydrogenation efficiency while addressing these challenges. The confinement effect of porous materials was shown to shift the thermodynamical equilibrium of several reactions,^18−20^ including CO_2_ hydrogenation, resulting in an increased yield of formic acid.^20^ It may be considered a cost-effective alternative to the transition metal catalysts due to (a) the higher density of the pore phase compared to the bulk phase, increasing yield for reactions in which there is a decrease in the total number of moles, by Le Chatelier’s principle,^21^ (b) the selective adsorption of favored HCOOH component on the solid surfaces, promoting its enhanced formation.^19^ In a molecular simulation study by Wasik et al.,^20^ the confinement effect of metal–organic frameworks (MOFs) was found to affect the CO_2_ hydrogenation reaction, shifting the thermodynamical equilibrium toward HCOOH formation. The most significant improvement in HCOOH production was achieved with Cu-BTC at 298.15 K and 60 bar, resulting in the mole fraction of HCOOH equal to 0.0016, which is ca. 2000 times higher compared to the gas phase.^20^ The final concentration of produced HCOOH (0.031 mol L^–1^^20^) was ca. 80 times lower than the highest reported concentration obtained with the use of Wilkinson complex,^16^ and ca. 30 times higher than the lowest reported concentration obtained using a catalyst with N-heterocyclic carbene ligands.^14^ By comparing the performance of MOFs with different pore sizes and metal centers (UiO-66, Cu-BTC, and IRMOF-1), it was found that the stronger confinement resulting from the smaller pore sizes does not ensure the enhancement in HCOOH production from the CO_2_ hydrogenation reaction. Despite the smallest pore sizes within the studied MOFs, the resulting mole fraction of HCOOH in the UiO-66 framework was only ca. 200 times higher compared to the gas phase at 298.15 K, 60 bar.^20^ The type of metal center in the metal–organic framework was found to be the dominant factor in HCOOH production.^20^ Metal–organic frameworks offer a promising alternative or supplement to transition metal catalysts for enhancing the efficiency of CO_2_ hydrogenation, as a low free energy barrier for the reaction compared to the gas phase is still needed to reach the favorable thermodynamic equilibrium in MOFs. This potential arises from several advantages: MOFs eliminate the need for expensive temperature elevation, produce a more valuable final product, which reduces the costs of downstream processing, and achieve a final concentration of HCOOH comparable to the reported concentrations of formate obtained with transition metal catalysts.^20^

The M-MOF-74 series allows to explore how the type of metal center impacts the enhancement of HCOOH production while minimizing the impact of pore size. The M-MOF-74 series (where M = Ni, Cu, Co, Fe, Mn, Mg, or Zn^22−30^) is one of the most popular families of MOFs, synthesized by combining M^2+^ ions with 2,5-dioxido-1,4-benzenedicarboxylate (dobdc^4–^) ligands. The presence of negatively charged ligands causes a high density of metal cations,^30^ accessible for sorbate molecules through large cylindrical pores with a diameter of ca. 11 Å.^30^ Numerous open-metal sites increase selectivity^31^ and the surface packing density of adsorbates.^32^ Additionally, the open-metal sites provide reactive sites for chemical reactions, such as oxygenation^33^ and size-selective Lewis acid catalysis.^34^ Extensive experimental and computational studies revealed the promising performance of the M-MOF-74 series for the capture and separation of CO_2_^30,35^ and H_2_.^22−27,29,36^ The solid adsorption using M-MOF-74 is considered an alternative to more expensive and less efficient liquid absorption.^37^ In the study of Queen et al.,^30^ the adsorption of CO_2_ in M-MOF-74 was analyzed experimentally, and computationally using density functional theory (DFT) calculations. It was found that depending on the open-metal site, the affinity of CO_2_ with M-MOF-74 frameworks decreases as follows: Mg > Ni > Co > Fe > Mn > Zn > Cu.^30^ The adsorption loading was obtained in the range from ca. 130 mg g^–1^ of framework for M = Cu to ca. 310 mg g^–1^ of framework for M = Mg at 100 kPa, 298 K, with the corresponding isosteric heat of adsorption from ca. 20 kJ mol^–1^ to ca. 40 kJ mol^–1^.^30^ The increase in isosteric heat of CO_2_ adsorption correlated to the stronger CO_2_ binding energy was found to result from a higher effective charge of the M^2+^ ion at the open-metal site where CO_2_ adsorbs. This implies that electrostatic interactions are the main factor affecting CO_2_ adsorption. In sharp contrast, H_2_ adsorption was found to be predominantly determined by polarization interactions at low temperatures,^28^ resulting in a difference in affinity with M-MOF-74 compared to CO_2_: Ni > Co > Mg > Fe > Zn ≈ Mn > Cu.^36^ The highest adsorption uptake of H_2_ is obtained for M = Ni as ca. 20 mg g^–1^ of framework at 100 kPa, 77 K.^36^ The study of Wasik et al.^38^ on the adsorption of CO_2_/H_2_ mixtures in M-MOF-74 showed that almost no adsorption of H_2_ occurs (less than 1 mg g^–1^ of H_2_ adsorbed) when CO_2_ is present in the mixture at 298.15 K. This suggests that H_2_ may be the limiting component in the CO_2_ hydrogenation reaction carried out in the confinement of M-MOF-74. Considering that both adsorbates CO_2_ and H_2_ show different affinities for adsorption in M-MOF-74, an interesting topic for research is to examine how the type of metal center in M-MOF-74 affects the adsorption of HCOOH obtained from the CO_2_ hydrogenation reaction.

Molecular simulations offer an intrinsic approach to explore confinement effects independently from catalytic effects, clarifying what is caused by confinement, and what is caused by catalysis. To the best of our knowledge, no literature data exists on force field-based molecular simulations of CO_2_/H_2_/HCOOH systems within the M-MOF-74 framework. In our previous work,^38^ we presented a nonpolarizable force field for molecular simulations of CO_2_ and H_2_ adsorption in M-MOF-74, where M = Ni, Cu, Co, Fe, Mn, Zn, after adjusting the existing force field for CO_2_, H_2_, and M-MOF-74 by scaling the Coulombic interactions of M-MOF-74 atoms to reproduce experimental data on CO_2_ adsorption,^30^ and scaling the Lennard–Jones interaction potentials between the center of mass of H_2_ and the open-metal centers to reproduce experimental data on H_2_ adsorption.^36^ The validation of the force field was confirmed by the successful reproduction of experimental CO_2_ and H_2_ adsorption isotherms, heats of adsorption, binding geometries, and demonstrating temperature transferability from 77 to 87 K, and 298 K. The advantages of a nonpolarizable force field adjusted to reproduce experimental data compared to a polarizable force field are easy transferability from one component to another, low computational time, and high accuracy.^38^ While polarizable force fields may have an improved description of interactions between guest molecules and open-metal sites,^39^ the computational cost is high unless back-polarization is ignored.^40^ A nonpolarizable force field for CO_2_ adsorption in M-MOF-74 was derived from DFT by Mercado et al.,^41^ but this approach involved adjusting not only the LJ interaction potentials of the metal site but also all interaction sites, leading to many fitting parameters and potentially lower transferability. Pham et al.^28^ attempted to reproduce experimental data on H_2_ adsorption in M-MOF-74^36^ by testing various models, among which only the polarizable model successfully reproduced the adsorption isotherms for all studied metal centers. For systems involving not only CO_2_ and H_2_ adsorption but also the hydrogenation reaction of CO_2_ to HCOOH, the size and complexity of the system can affect computational time and accuracy. Developing a nonpolarizable force field is beneficial for investigating the dependence of HCOOH production enhancement on the type of metal center in M-MOF-74. Additionally, nonpolarizable force fields offer the advantage of transferability between different components, whereas polarizable force fields require specific development for transferability.^28,40^ In this work, molecular simulations were used to study the adsorption and production of HCOOH from the CO_2_ hydrogenation reaction in M-MOF-74, where M = Ni, Cu, Co, Fe, Mn, Zn. Due to the lack of experimental data available for the adsorption of formic acid in M-MOF-74, the compatibility of the nonpolarizable force field for CO_2_ and H_2_ adsorption in M-MOF-74^38^ with HCOOH force field^42^ was evaluated by studying the binding geometries of HCOOH, using both a minimization scheme and DFT. Monte Carlo simulations in the grand-canonical ensemble (GCMC) were performed in the frameworks to compute single-component adsorption isotherms of HCOOH and the adsorption isobars of the CO_2_ hydrogenation components. The effect of the type of metal center in M-MOF-74 on the CO_2_ hydrogenation reaction was studied at less industrially expensive temperatures ranging from 298.15 to 800 K and higher pressures from 1 to 60 bar, which allow more molecules to enter the structure.

This manuscript is organized as follows: in Section 2, we provide technical details of the molecular simulation methods, the force fields for CO_2_, H_2_, HCOOH, and the metal–organic frameworks. In Section 3, we present and discuss the results. The HCOOH isotherms and heat of adsorption in M-MOF-74 are computed using GCMC simulations at 298 K, and 10^–6^–10 kPa. The binding geometries are simulated using Baker’s minimization scheme^43^ and compared to the results of DFT calculations. The adsorption isobars in M-MOF-74 frameworks are computed from GCMC simulations at 298.15–800 K and 1–60 bar, using gas-phase mole fractions of CO_2_, H_2_, and HCOOH at reaction equilibrium, obtained in our previous work.^44^ The HCOOH production enhancement is calculated for all systems. Depending on the metal center, the enhancement in HCOOH production decreases in the same order as its uptake and isosteric heat of adsorption: Ni > Co > Fe > Mn > Zn > Cu. The strongest guest–host interaction of HCOOH with Ni-MOF-74 causes the most significant influence on the CO_2_ hydrogenation thermodynamics, enhancing HCOOH production by ca. 10^5^ times compared to the gas phase at 298.15 K, 60 bar. Our findings are summarized in Section 4.

## 2 Methodology

The adsorption of HCOOH and the CO_2_ hydrogenation reaction is studied in M-MOF-74, where M = Ni, Cu, Co, Fe, Mn, Zn using force field-based molecular simulations. Intermolecular interactions between guest–host and guest–guest molecules are modeled using Coulombic and Lennard–Jones (LJ) potentials. The Lorentz–Berthelot mixing rules^45^ are used for interactions between different LJ sites, except interactions between the H_2_ molecule centers of mass and open-metal centers, which are scaled and specified by an override.^38^ LJ interactions are cut and shifted to zero at a 12 Å cutoff radius without tail corrections. Periodic boundary conditions are applied in all three directions. Electrostatic interactions are computed using the Ewald summation method,^46^ with parameters corresponding to a relative precision of 10^–6^. The so-called “P2” variant of the OPLS/AA force field for HCOOH^42^ is applied to the HCOOH molecule model constructed and optimized at the B3LYP/6-31G(d) level of theory in a study of Wasik et al.^44^ The HCOOH force field successfully reproduce the vapor–liquid equilibrium coexistence curve, saturated vapor pressures, and densities at different temperatures.^44^ The nonpolarizable CO_2_, and H_2_ force field for adsorption in M-MOF-74 was adjusted in our previous work^38^ by introducing two modifications to the existing parameters for CO_2_, H_2_, and M-MOF-74: (1) Coulombic interactions of M-MOF-74 were scaled to reproduce experimental data on CO_2_ adsorption^30^ using the CO_2_ model by Harris and Yung^47^ combined with the LJ interaction parameters modeled by García-Sánchez et al.,^48^ (2) LJ interaction potentials between the center of mass of H_2_ in the three-site charge-quadrupole model by Darkrim–Levesque model,^49^ and the open-metal centers were scaled to reproduce experimental data on H_2_ adsorption.^36^ The LJ parameters for the framework atoms are derived from the DREIDING force field,^50^ except for the metal centers, which use parameters from the UFF force field.^51^ The CO_2_, H_2_, HCOOH, and framework models are rigid, with point charges assigned to all atoms. All framework structures are charge-neutral. All the studied M-MOF-74 crystal structures were obtained from experimental syntheses.^22−27^ The simulated systems consist of 1 × 1 × 4 trigonal unit cells to guarantee a minimum distance that exceeds twice the cutoff radius between periodic images. The LJ parameters and partial charges for all components used in this work are listed in Table S1 of the Supporting Information. For the details on the structures, Lennard-Jones and Coulombic interaction potentials for the M-MOF-74 (M = Ni, Cu, Co, Fe, Mn, Zn) frameworks, the reader is referred to the study of Wasik et al.^38^

The adsorption isotherms and the heat of HCOOH adsorption in M-MOF-74 were computed from GCMC simulations^52^ at 298 K, 10^–6^–10 kPa, using RASPA software package.^53,54^ In the grand-canonical ensemble, the chemical potential, volume, and temperature are fixed. The RASPA software package^53^ provides the uncertainties in the computed number of molecules adsorbed in a unit cell, by dividing the simulation into five blocks and calculating the standard deviation. The heat of HCOOH adsorption at finite loadings was computed using the fluctuation method^55^ implemented in the RASPA software package.^53^ To evaluate the resulting adsorption of HCOOH, we studied the interactions between an adsorbate molecule and the frameworks. The isosteric heat of adsorption^56^ for HCOOH in M-MOF-74 was calculated for a temperature range of 298.15–800 K, and compared with literature data for the previously studied MOFs (UiO-66, Cu-BTC, IRMOF-1).^20^ The enthalpy of adsorption at infinite dilution representing the affinity between the molecule and the framework, is determined by^57^
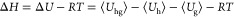
1where Δ*U* is the internal energy of the system, ⟨*U*_hg_⟩ is the average energy of the guest molecule in the host framework, ⟨*U*_h_⟩ is the average energy of the host framework (0 J for rigid frameworks), ⟨*U*_g_⟩ is the average energy of the guest molecule (0 J for rigid molecules), *R* is the universal gas constant, and *T* is the temperature.

To analyze the binding geometries of the adsorption of formic acid at infinite dilution, we performed a series of geometry optimizations of a single molecule using Baker’s minimization method^43^ implemented in RASPA.^53,54^ Baker’s minimization method uses the eigenvalues/vectors of the Hessian matrix to efficiently and accurately locate true minima on the energy surface, ensuring the determination of equilibrium geometries with enhanced numerical stability and faster convergence.^43^ Because of the asymmetric nature of formic acid, we performed 100 optimizations starting from different configurations and ranked the optimized geometries from high to low energy. The obtained equilibrium geometries of HCOOH were compared with DFT calculations. The adsorption of formic acid in M-MOF-74 has been performed using DFT and plane wave pseudo potential method (PWSCF) implemented in the Quantum Espresso package.^58^ The exchange-correlation corrections has been applied using the generalized gradient approach (GGA), as formulated by Perdew, Burke and Ernzerhof (PBE),^59^ including DFT-D3(BJ) dispersion corrections.^60^ The kinetic energy cutoff for wave functions has been set to 60 Ry, while the kinetic energy cutoff for charge density and potential, using norm-conserving pseudopotentials, has been set to 480 Ry. The unit cell of M-MOF-74 includes 54 atoms (see Figure S1 of the Supporting Information) and has been fully relaxed by allowing both the ionic positions and lattice parameters to change until the convergence threshold for the total energy and forces are smaller than 1 × 10^–6^ a.u. Due to the presence of transition metals (Co, Cu, Fe, Mn, Ni, and Zn) in M-MOF-74, the smearing method has been selected to handle the electronic occupations of the Kohn–Sham states, using a degauss value of 1.4 × 10^–2^ Ry. Since M-MOF-74 includes atoms with magnetic properties, appropriate magnetization values have been considered depending on the transition metal. To ensure accurate results, a convergence threshold of 1 × 10^–9^ a.u. has been selected with a mixing β of 0.4. The Brillouin zone has been sampled using a 2 × 2 × 2 Monkhorst–Pack *k*-points.^61^ The calculated lattice parameters resulting from the geometry optimization show a strong agreement with the previously reported literature^30,62^ (see Figure S2 of the Supporting Information). To study the adsorption of formic acid in the M-MOF-74 family, we have performed a geometry relaxation of a single molecule within the previously optimized structures. In this calculation, both MOF and formic acid molecule are relaxed. Finally, the binding energy between formic acid and the surface of M-MOF-74 has been computed by
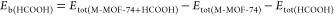
2where *E*_tot(M–MOF–74+HCOOH)_ indicates the total energy of M-MOF-74 with HCOOH per unit cell, *E*_tot(M–MOF–74)_ denotes the total energy of M-MOF-74 per unit cell, and *E*_tot(HCOOH)_ represents the total energy of isolated formic acid molecule.

To study the thermodynamic confinement effects of M-MOF-74 on the CO_2_ hydrogenation to HCOOH, the adsorption isobars were computed using GCMC simulations at 298.15–800 K and 1–60 bar. The gas-phase mole fractions of CO_2_, H_2_, and HCOOH at chemical equilibrium, obtained using the Monte Carlo Software Brick-CFCMC^63,64^ from Continuous Fractional Component Monte Carlo simulations^65−67^ in the reaction ensemble^68−70^ (Rx/CFC) by Wasik et al.,^20^ served as input data for the GCMC simulations. The chemical potential is directly derived from the fugacity, which is calculated using the fugacity coefficients from the Peng–Robinson equation of state (PR-EoS)^71^ by the RASPA software package.^53,54^ The agreement between the fugacity coefficients of CO_2_, H_2_, and HCOOH computed using the PR-EoS and the NIST Standard Reference Database REFPROP^72^ at 298.15–800 K and 1–60 bar was found to be satisfactory, with average deviations of only 0.45% for CO_2_ and 0.66% for H_2_, as reported in the study of Wasik et al.^20^ The initial mole fractions^20^ used in this study are listed in Table S2 of the Supporting Information. The uncertainties in the computed number of molecules adsorbed in a unit cell provided by the RASPA software package^53^ were used to calculate the uncertainties in the mole fractions of components *Err*_*x*_ by
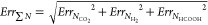
3
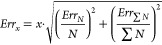
4where *Err*_∑*N*_ is the uncertainty of the total number of molecules of all components adsorbed in a unit cell, *Err*_*N*_ is the uncertainty of the computed number of molecules adsorbed in a unit cell, *x* is a mole fraction of the component, and *N* is the computed number of molecules of the component. To compare the mole fractions of HCOOH obtained from GCMC simulations in the frameworks to the gas-phase mole fractions at equivalent chemical potentials,^20^ the enhancement (ENH) of HCOOH production was calculated as
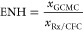
5where *x*_GCMC_ and *x*_Rx/CFC_ are mole fractions of HCOOH resulting from GCMC and Rx/CFC simulations,^20^ respectively. The final concentration of HCOOH in the adsorbed phase was calculated as
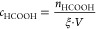
6where *n*_HCOOH_ is the calculated number of moles of HCOOH adsorbed in a unit cell, ξ is the helium void fraction, and *V* is the volume of the unit cell.

The GCMC simulations for HCOOH adsorption in M-MOF-74 were conducted with 10^4^ initial Monte Carlo (MC) cycles followed by 10^6^ production MC cycles. Each MC cycle consists of *N* trial moves, where *N* is the total number of molecules at the beginning of the simulation. The probabilities assigned to different trial moves in these GCMC simulations were 25% for translations, 25% for rotations, 25% for reinsertions, and 25% for swap trial moves (exchanging molecules with the reservoir). For the GCMC simulations of the CO_2_ hydrogenation reaction to HCOOH, 10^4^ equilibration MC cycles, and 4 × 10^5^ production MC cycles were used. The probabilities for these trial moves were 16.7% translations, 16.7% rotations, 16.7% reinsertions, 16.7% identity changes (changing the identity of a selected molecule), and 33.2% swap trial moves. For further details on Monte Carlo trial moves, see refs (53,54,73).

## 3 Results and Discussion

**Figure 1:**
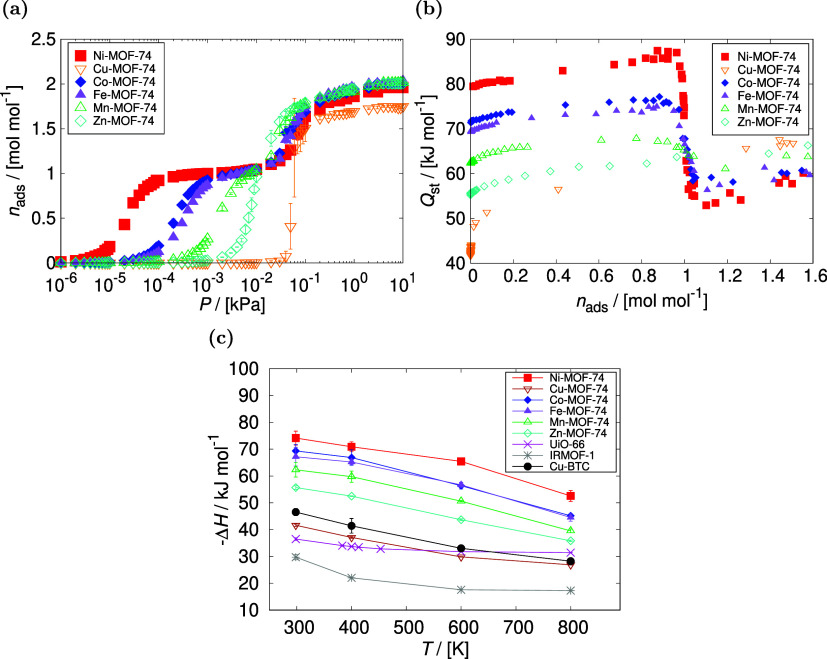
Adsorption of HCOOH in M-MOF-74 (M = Ni, Cu, Co, Fe, Mn, Zn) computed from GCMC simulations: (a) the adsorption isotherms at 298 K, and 10^–6^–10 kPa, (b) the corresponding heat of adsorption at 298 K, and 10^–6^–10 kPa, and (c) the isosteric heat of adsorption at 298.15–800 K. The units of adsorption loading [mol mol^–1^] refer to the number of HCOOH molecules adsorbed per metal center. The trend in the simulated uptake and heat of HCOOH adsorption depends on the open-metal site as follows: Ni > Co > Fe > Mn > Zn > Cu.

To investigate the adsorption of HCOOH in M-MOF-74 (M = Ni, Cu, Co, Fe, Mn, Zn), we first computed the isotherms and heats of adsorption for all the studied frameworks at 298 K, and 10^–6^–10 kPa, see Figure 1a,b. At low pressures, a significant variation in HCOOH adsorption characteristics is observed, indicating that the open-metal sites have a dominant influence on the adsorption process. The HCOOH molecules start to fill the Ni-MOF-74 structure at the lowest pressure, ca. 10^–5^ kPa with the corresponding heat of adsorption ca. 80 kJ mol^–1^. Cu-MOF-74 starts to fill at the highest pressure, ca. 4 × 10^–2^ kPa with the corresponding heat of adsorption ca. 40 kJ mol^–1^, followed by rapid nucleation of adsorbate molecules. The trend in the simulated uptake and heat of HCOOH adsorption depends on the open-metal site as follows: Ni > Co > Fe > Mn > Zn > Cu. The same trend is found for the adsorption of CO_2_ in M-MOF-74, which suggests that HCOOH adsorption is also predominantly influenced by the electrostatic interactions dependent on the effective charge of the M^2+^ ion at the open-metal site. The two-step mechanism of adsorption, wherein the adsorbate molecules first adsorb at the metal centers, followed by adsorption above a triangle of oxygen atoms within the framework, is present in the adsorption isotherms of Ni-, Co-, Fe-, and Mn-MOF-74 frameworks. The primary adsorption sites fill until ca. 1 molecule of HCOOH per metal center, resulting in a sudden decrease in the heat of adsorption when the secondary sites start filling. While the binding affinities at the primary adsorption sites differ within the M-MOF-74 series, the isotherms converge as the metal centers become saturated at higher pressures due to the isostructural properties of the frameworks. As pressures of 1 kPa, the capacities of the Ni-, Co-, Fe-, Mn-, and Zn-MOF-74 frameworks become highly comparable, each accommodating ca. 2 molecules of HCOOH per metal center. The dependence of HCOOH affinity in M-MOF-74 on a temperature was investigated in the range from 298.15 to 800 K and compared with literature data for different MOFs.^20^ In Figure 1c, the isosteric heat of adsorption for HCOOH is shown, which is a measure of the change in enthalpy when adsorbate molecules are adsorbed from the gas phase (higher energy state) to the adsorbed phase (lower energy state), causing the release of heat. The energy state of HCOOH on the adsorbent surface increases with temperature, leading to weaker interactions between the framework and adsorbate. The isosteric heat of adsorption corresponds to the values obtained from GCMC simulations of adsorption at low pressure, where the adsorption loading is very low. The affinity of HCOOH in MOFs decrease with the isosteric heat of adsorption in the following order: Ni-MOF-74 > Co-MOF-74 > Fe-MOF-74 > Mn-MOF-74 > Zn-MOF-74 > Cu-BTC > Cu-MOF-74 > UiO-66 > IRMOF-1. The strongest isosteric heat of adsorption was found for Ni-MOF-74, resulting in the isosteric heat of adsorption ca. 75 kJ mol^–1^ at 298.15 K. The obtained value is 1.6 times higher than the isosteric heat of adsorption in Cu-BTC (ca. 45 mol^–1^), the best-performing MOF for CO_2_ hydrogenation reaction from our previous study.^20^ This indicates that Ni-MOF-74 can be expected to be a more promising candidate for CO_2_ hydrogenation application, than Cu-BTC which was found to enhance HCOOH production ca. 2000 times compared to the gas phase.^20^

**Figure 2:**
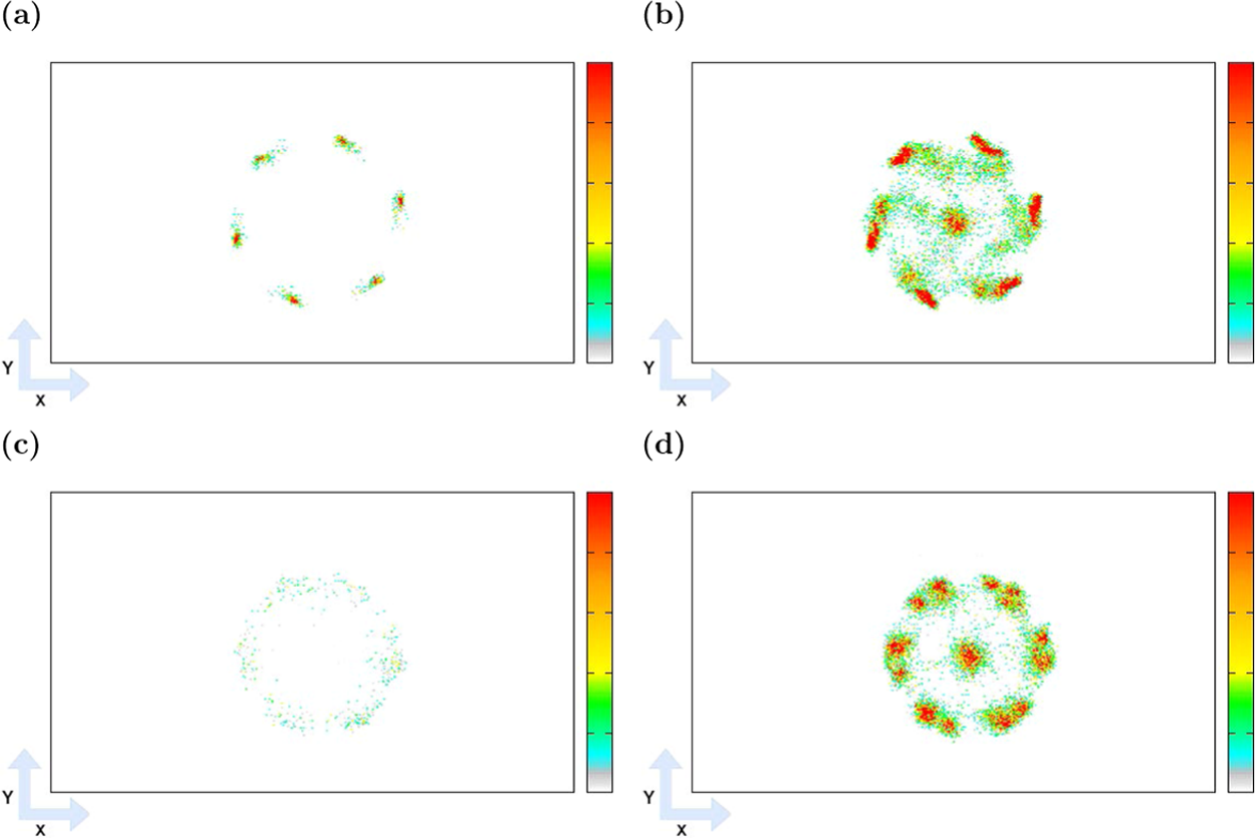
Distribution of the HCOOH molecules inside M-MOF-74, analyzed using density profiles from GCMC simulation: (a) Ni-MOF-74 at 298 K, 10^–5^ kPa, (b) Ni-MOF-74 at 298 K, 10 kPa, (c) Cu-MOF-74 at 298 K, 4 × 10^–2^ kPa, (d) Cu-MOF-74 at 298 K, 10 kPa. The center of mass of the molecules that are adsorbed was projected onto the *XY* plane. The color gradation of the scales relates to the most and least populated regions of the structure, which is relative in each case. The color scale is shown as a reference for the molecule loading. The preferential sites of HCOOH molecules (colored red) in Ni-MOF-74 are at the open-metal centers. The adsorption loading of HCOOH in Cu-MOF-74 is more homogeneously distributed, which is a reflection of the lower affinity for Cu.

The distribution of HCOOH molecules was analyzed inside M-MOF-74 using the average density profiles and shown for Ni-, and Cu-MOF-74 in Figure 2. The center of mass of the adsorbed molecules was projected onto the *XY* plane of anisotropic frameworks. The average density profiles of HCOOH in Ni-MOF-74 confirm that the open-metal centers are primary adsorption sites, where molecules adsorb at low pressures. As the adsorption proceeds at higher pressures, the molecules also adsorb above a triangle of oxygen atoms within the framework. A very high adsorption loading is observed at the open-metal centers compared to the secondary adsorption sites. The adsorbed molecules of HCOOH in Cu-MOF-74 are more homogeneously distributed, due to the lowest affinity for Cu-MOF-74 among the studied structures and the lack of a two-step mechanism of adsorption. The distribution of HCOOH molecules inside Co-, Fe-, Mn-, and Zn-MOF-74 is shown in Figure S3 of the Supporting Information.

**Figure 3:**
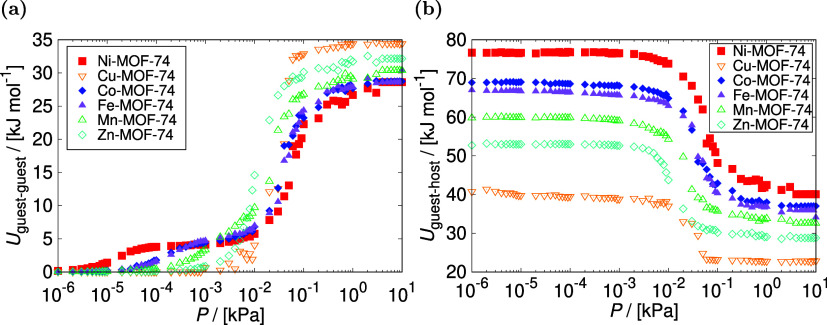
Interaction energies of HCOOH in M-MOF-74 (M *=* Ni, Cu, Co, Fe, Mn, Zn) computed from GCMC simulations at 298 K, and 10^–6^–10 kPa: (a) guest–guest interaction energies, (b) guest–host interaction energies. The increase in guest–guest interaction energies with pressure indicates that the adsorption mechanism at the secondary adsorption sites is driven by the nucleation of HCOOH molecules through hydrogen bond interactions. As the adsorption loading increases in the frameworks, guest–host interaction energies decrease as the preferential adsorption sites fill up, and guest–guest interactions become more important.

The guest–guest interaction energies were analyzed and shown in Figure 3a as a function of the pressure. The increase in guest–guest interaction energies with pressure indicates that the adsorption mechanism at the secondary adsorption sites is driven by the nucleation of polar HCOOH molecules via hydrogen bond interactions. The largest jump in the guest–guest interaction energy is observed for Cu-MOF-74 which reflects the rapid nucleation of adsorbate molecules shown in adsorption isotherm at the pressure range from 4 × 10^–2^ kPa to 10^–1^ kPa. At the saturation pressure of 10 kPa, the guest–guest interaction energies for all the studied MOFs are close to the enthalpy of vaporization for HCOOH, which is reported as ca. 20.1 kJ mol^–1^ at 298.15 K^74^ or 29.6 kJ mol^–1^ at 303 K.^75^ The energy contribution from the interaction between HCOOH and the adsorbents is shown in Figure 3b. The affinity between the adsorbate and the framework increases with the guest–host interaction energy. As the loading increases with pressure, guest–host interactions weaken due to the preferential adsorption sites filling up, and guest–guest interactions becoming more significant. The guest–host interactions at a low-pressure regime correspond to the isosteric heat of adsorption in infinite dilution.

**Figure 4:**
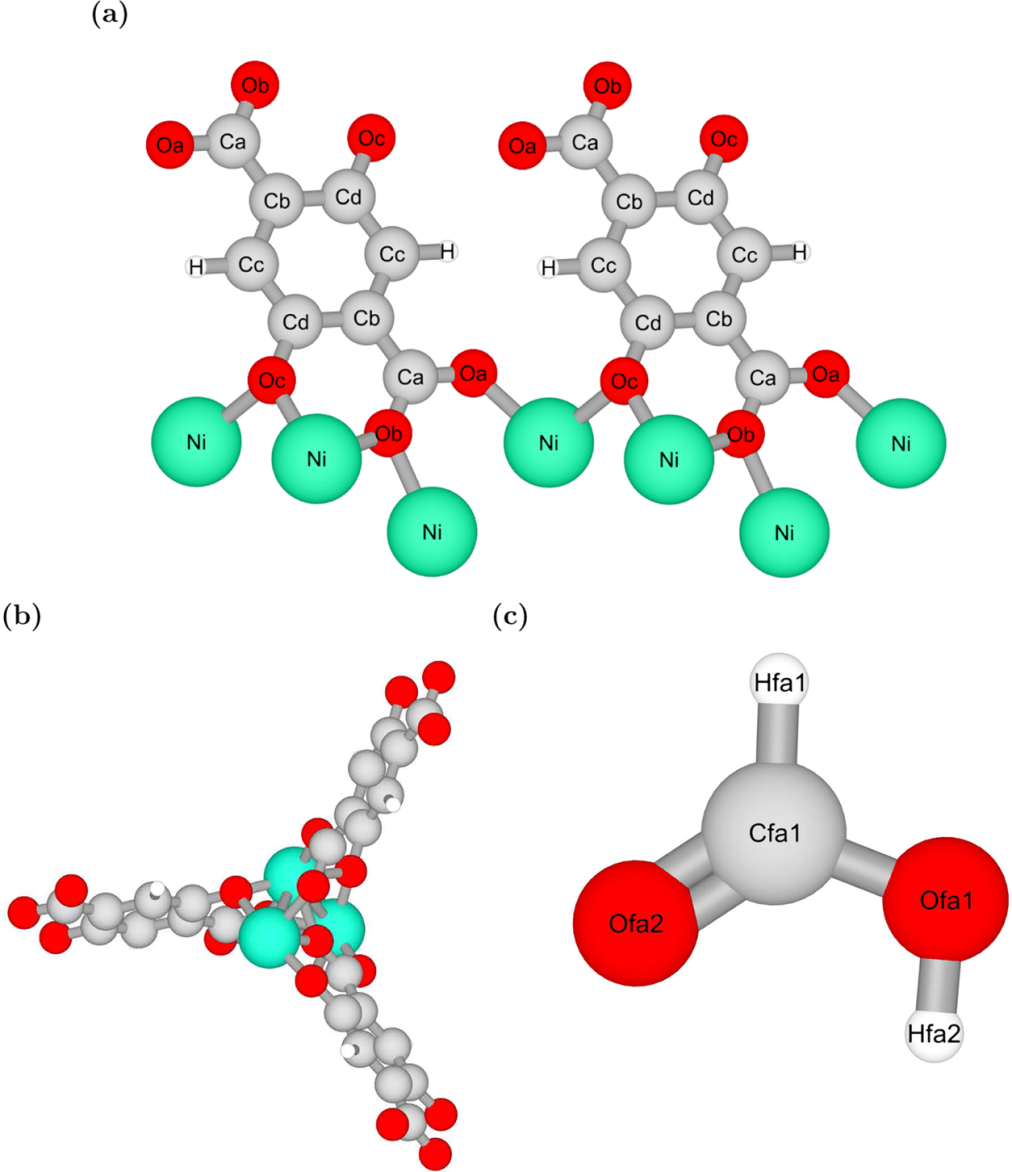
Schematic representation visualized using iRASPA^73^ of: (a) the labels and positions of different types of atoms in the M-MOF-74 frameworks for the example of Ni-MOF-74, (b) an atomistic reference structure of MOF-74 framework, and (c) HCOOH model with the atoms labeled.

The binding geometries of the HCOOH adsorption were analyzed using Baker’s minimization method^43^ and ranked from high to low energy to find the favorable configurations. To provide a clear depiction of the atomic positions within the frameworks and the HCOOH molecule, a schematic representation of Ni-MOF-74 is shown in Figure 4 together with the HCOOH model.

**Figure 5:**
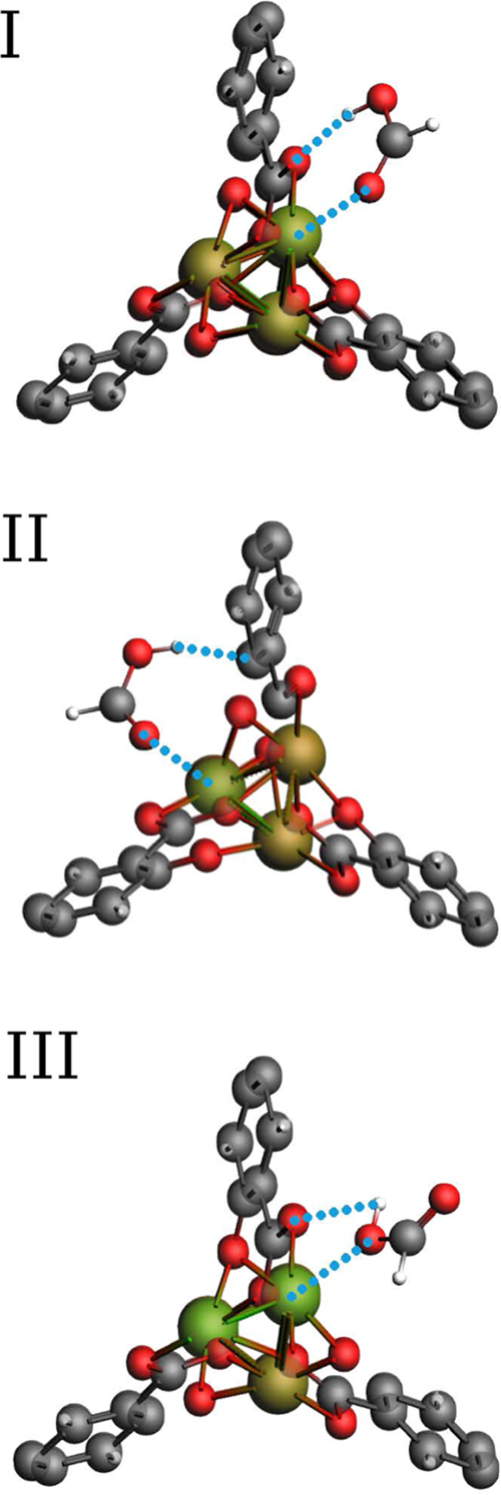
Primary binding geometries of the HCOOH adsorption in M-MOF-74 (M = Co, Cu, Fe, Mn, Ni, and Zn) obtained using Baker’s minimization method.^43^ In the most stable configuration I, the O_fa2_ atom of HCOOH molecule points to the metal center while the H_fa2_ aligns to the Oa atoms in the metal cluster. In configuration II, the O_fa2_ atom of HCOOH points to the metal and the H_fa2_ points to the Cb carbon atom of the aromatic ligand. In the least favorable configuration (configuration III), the OH group of HCOOH is placed in a parallel configuration concerning the M-Oa bond of the metal cluster, while the O_fa2_ atom points to the center of the cage.

The three primary binding geometries of HCOOH found in the surroundings of the metal centers are shown in Figure 5. In the three situations, one of the oxygen atoms of HCOOH and the hydrogen atom of the hydroxyl (OH) group strongly interact with the metal center and a negatively charged atom close to it. Configuration I is the most stable, followed by configurations II and III. In configuration I, the electronegative O_fa2_ atom of HCOOH molecules points to the metal while the H_fa2_ aligns to one of the oxygen atoms in the metal cluster (Oa). Unlike the other two oxygen atoms (Ob and Oc) of M-MOF-74 that are connected to two metal atoms, the Oa oxygen atom is connected to a single metal atom and to a carbon atom (Ca). As a result, it can act as a hydrogen bond acceptor, while the OH group of HCOOH is a hydrogen bond donor group. Because of this combined interaction, the configuration I shows the highest binding energy compared to the other two configurations. The orientation of the molecule in this configuration is driven by the electrostatic field lines within the cavities of these MOFs, which go from the positively charged metal sites to the electronegative Oa atoms,^76^ see Figure S4 of the Supporting Information. Similarly to configuration I, in configuration II, the electronegative O_fa2_ atom of HCOOH points to the metal, but in this case, the OH group points to the Cb carbon atom of the aromatic ligand, which has a negative charge. In the less favorable configuration (configuration III), the OH group of HCOOH is nearest to the metal center, while the O_fa2_ atom points to the center of the cage. In this configuration, the OH group is placed in a parallel configuration concerning the M-Oa bond of the metal cluster. To provide a better understanding of the HCOOH binding configurations, the Coulombic potentials for M-MOF-74 were normalized with respect to the partial charge of the metal center, see Table S3 of the Supporting Information. The calculated relative charges exhibit a high degree of similarity among the different atom types identified across all frameworks. Normalization reveals that in the most stable configuration I, H_fa2_ aligns with the available hydrogen bond acceptor Oa of the strongest electronegativity (relative charge of −0.40). In the following configuration II, H_fa2_ points to the next in order of electronegativity Cb carbon atom (−0.15). The analysis suggests that the electronegativity of the ligand atom significantly affects the binding configuration of HCOOH. Specifically, together with the open metal sites, atoms with stronger electronegativity are identified as the primary sites for HCOOH adsorption.

**Figure 6:**
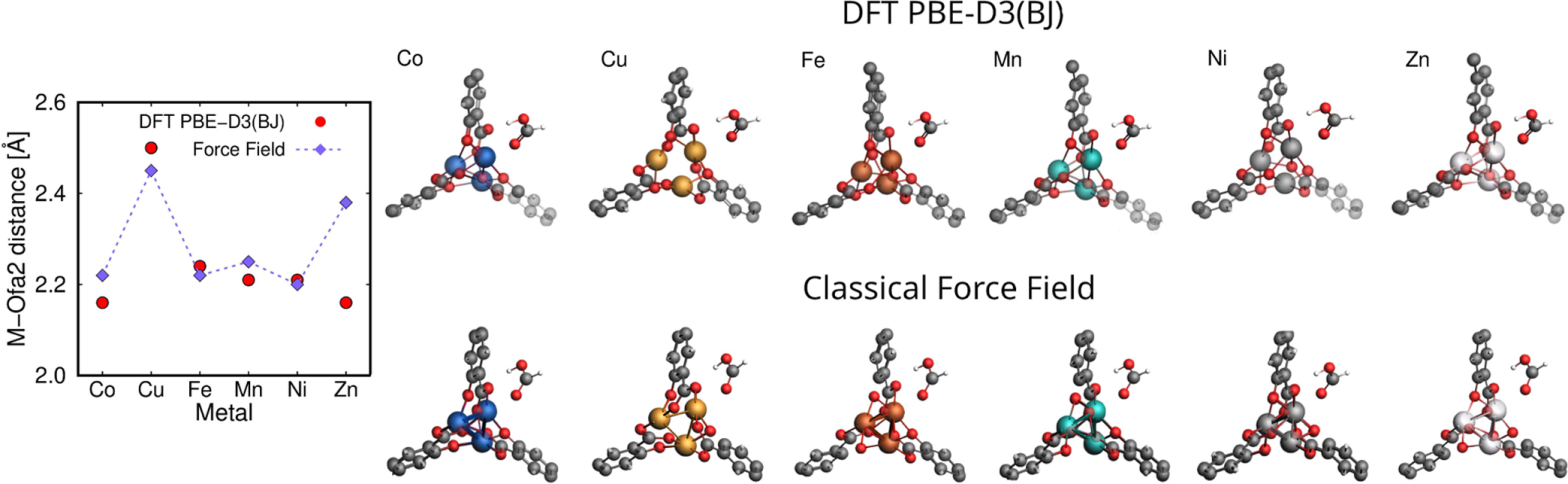
Binding geometries of the most stable configuration I of HCOOH in M-MOF-74 (M = Co, Cu, Fe, Mn, Ni, and Zn) obtained from force field-based molecular simulations compared to the DFT (PBE-D3(BJ)) results. The resulting distances between O_fa2_ atom of HCOOH molecule and the metal center are plotted for all the framework types. The optimized geometries of HCOOH calculated by DFT (top) are visualized with the geometries computed from the force field-based simulations (bottom) for comparison. The general trend of distances is similar across both methods, except Zn-MOF-74 due to its highest reactivity. The lines connecting the symbols are used to guide the eye.

The binding geometries and the M-O_fa2_ distances of the most stable configuration I of HCOOH in M-MOF-74 obtained from force field-based molecular simulations were compared with the DFT (PBE-D3(BJ)) results in Figure 6. The distances between O_fa2_ atom of HCOOH molecule and the metal center obtained from both methods differ by no more than 0.1 Å for all the studied frameworks, except Zn-MOF-74. The slightly higher discrepancy of ca. 0.2 Å between the distances computed from force field-based molecular simulations and DFT could result from the highest reactivity of Zn metal center,^77^ which is not accounted for in the classical force field. Within the M-MOF-74 family, Zn-MOF-74 was found to exhibit the highest catalytic activity toward several reactions, e.g., water dissociation,^78^ CO_2_ cycloaddition reaction with epoxides,^79^ and HCOOH synthesis via CO hydration.^80^ The obtained binding distances in all the frameworks range from approximately 2.15 to 2.5 Å. The closest configuration of HCOOH to the metal center is found in Ni-MOF-74 according to force field-based molecular simulations, and in Co-MOF-74 based on DFT. The farthest configuration is found in Cu-MOF-74 according to both methods. The binding energies of the most stable configuration I of HCOOH in M-MOF-74 obtained from force field-based molecular simulations and the DFT (PBE-D3(BJ)) calculations are shown in Figure S5 of the Supporting Information. The binding energies vary between the methods but follow a similar general trend across the different metal centers. Both computational methods indicate that Cu-MOF-74 has the weakest binding affinity, while Ni-MOF-74 shows the strongest binding affinity. It is worth mentioning that the binding geometries of HCOOH calculated with the classical force field were obtained using rigid frameworks, while in the DFT calculations, the molecule and framework atoms, as well as the system volume were allowed to relax. Despite these different approaches that could substantially affect the binding geometries and energies, Figures 6 and S5 of the Supporting Information show a reasonable agreement between DFT calculations and classical simulations. The relative difference between the two methods is 3% (or 1.6% excluding highly reactive Zn-MOF-74) for binding geometries and 18% for binding energies. This confirms the validity of the force field to describe the complex interactions between HCOOH and M-MOF-74, and no adjustments of the existing force field for HCOOH are needed. In cases where the binding geometries would show high deviations between the two computational methods, the adjustment of the force field by scaling the Lennard-Jones interaction potentials and/or the Coulombic potentials is necessary. The binding distance between the metal center of the MOF and the atoms of the adsorbate molecule can be modified by applying a scaling factor to σ (the distance at which the intermolecular potential between the two particles is zero). To modify the binding energy of the adsorbate in MOF, ϵ (the depth of the potential well) and/or partial charges *q* should be adjusted by applying a scaling factor.

**Figure 7:**
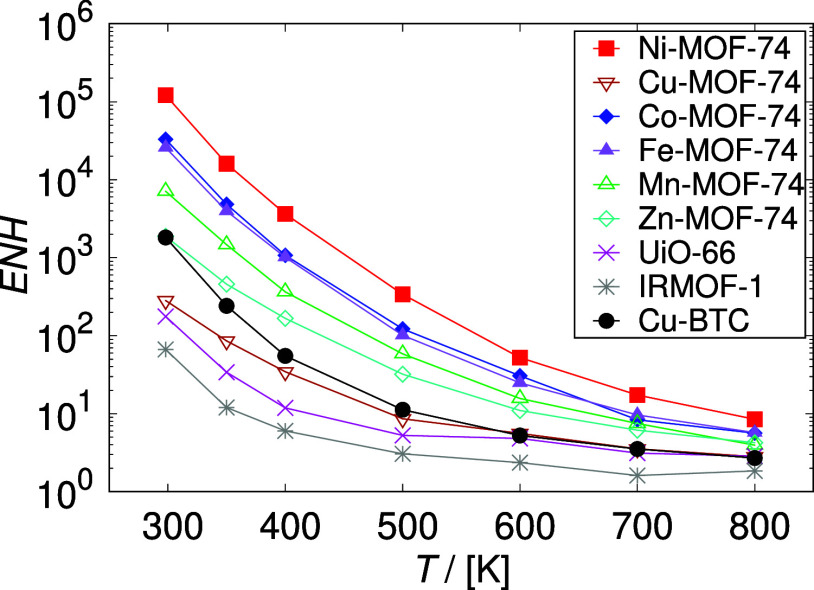
Enhancement (ENH, eq 5) in the production of HCOOH obtained from GCMC simulations in M-MOF-74 frameworks at 298.15–800 K and 60 bar, compared with the literature data for Cu-BTC, UiO-66, and IRMOF-1.^20^ The enhancement in HCOOH production decreases with increasing temperature due to the weakening of guest–host interactions. The performance of M-MOF-74 in the production of HCOOH in confinement follows the same order as the uptake and the heat of HCOOH adsorption: Ni > Co > Fe > Mn > Zn > Cu. The enhancement in the HCOOH production is the highest in the Ni-MOF-74 framework at 298.15 K, resulting in the mole fraction of HCOOH being ca. 10^5^ times higher compared to the gas phase.

The process of CO_2_ hydrogenation to HCOOH using M-MOF-74 is promising as the molecules of HCOOH are found to interact strongly with the frameworks, especially with Ni-MOF-74, resulting in high adsorption loadings. The mole fractions obtained from GCMC simulations for M-MOF-74 frameworks are provided in Tables S4–S9 of the Supporting Information. Figure S6 of the Supporting Information shows a comparison of HCOOH mole fractions obtained in M-MOF-74 and the literature data for Cu-BTC, UiO-66, and IRMOF-1^20^ at 298.15–800 K and 60 bar. The increase in pressure raises the concentration of HCOOH molecules, driving more molecules into the M-MOF-74 structure, filling its pores, and ensuring the system reaches a new equilibrium state with higher adsorbate loading by Le Chatelier’s principle.^21^ The mole fractions of HCOOH decrease with increasing temperature due to weakening guest–host interactions. The optimal conditions for all the systems, resulting in the highest mole fraction of HCOOH, are found at 298.15 K and 60 bar. The enhancement in HCOOH production due to the confinement within M-MOF-74 was calculated and compared with the literature data for Cu-BTC, UiO-66, and IRMOF-1^20^ at 298.15–800 K and 60 bar, see Figure 7. The performance of M-MOF-74 in the production of HCOOH in confinement follows the same order as the uptake and the heat of HCOOH adsorption: Ni > Co > Fe > Mn > Zn > Cu. The application of Ni-MOF-74 framework resulted in the highest enhancement of HCOOH production. The obtained mole fraction of HCOOH reaches ca. 0.1 at 298.15 K, which is ca. 10^5^ times higher compared to the gas phase. There is a significant difference in the enhancement of HCOOH production between Ni-MOF-74 and the second best-performing framework Co-MOF-74, which application results in *x*_HCOOH_ = 33,000 times higher than in the gas phase. The enhancement in HCOOH production using Ni-MOF-74 is ca. 60 times higher than the enhancement achieved with Cu-BTC in our previous work (mole fraction of HCOOH obtained with Cu-BTC was ca. 2000 times higher compared to the gas phase).^20^ Interestingly, the confinement effect of Cu-MOF-74 was found to be ca. 7 times weaker than that of Cu-BTC, resulting in the mole fraction of HCOOH ca. 300 times higher compared to the gas phase.

To assess what has the largest impact on HCOOH production using MOFs with the same type of metal centers, radial distribution functions were computed for Cu-MOF-74 and Cu-BTC at 298.15 K, see Figure S7 of the Supporting Information. While ca. 6 molecules are present in Cu-BTC within the preferential distance to the metal centers of 2.4 Å,^20^ in Cu-MOF-74 only ca. 1.5 adsorbed molecules of HCOOH are within the distance of 2.54 Å to the metal centers. This difference in intensity is due to the higher charge on the metal center in Cu-BTC compared to Cu-MOF-74. Another reason for the higher affinity of HCOOH in Cu-BTC than Cu-MOF-74 is the presence of different types of ligands in the structures. By comparing the intensity of HCOOH adsorption in configuration pointing to the most electronegative ligand atom of the framework, it was found that the stronger electronegativity of the oxygen atom in Cu-BTC leads to a higher intensity of HCOOH adsorption oriented toward this atom (H_fa2_-O1) that in Cu-MOF-74 where the oxygen atom is less electronegative (H_fa2_-Oa). The affinity with the framework also affects the formation of hydrogen bonds between HCOOH molecules. The HCOOH nucleation and dimerization are slightly more intense in Cu-MOF-74 than in Cu-BTC due to weaker interactions with the framework.

**Figure 8:**
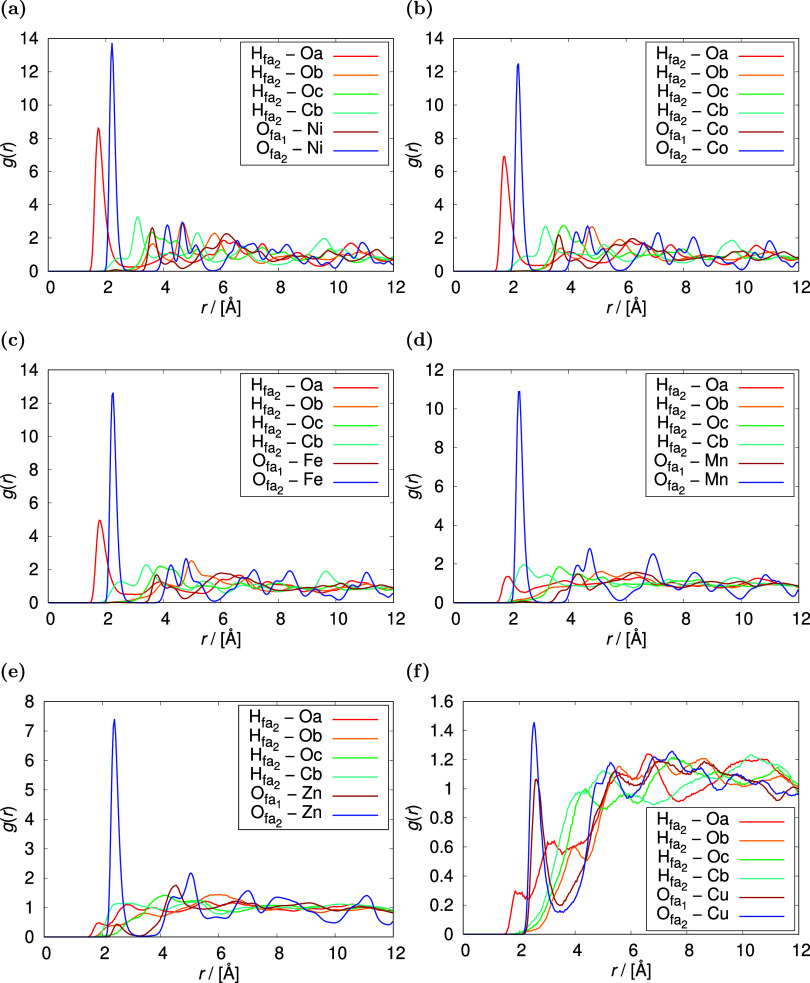
Radial distribution functions simulated for 50 molecules of HCOOH at 298 K in (a) Ni-MOF-74, (b) Co-MOF-74, (c) Fe-MOF-74, (d) Mn-MOF-74, (e) Zn-MOF-74, and (f) Cu-MOF-74. The presence of three primary binding geometries of HCOOH found in the surroundings of the metal centers is confirmed. In Cu-MOF-74, which has the weakest affinity with HCOOH, configuration III is found to outperform configuration II in terms of stability. The simulation was performed using the RASPA software package.^53,54^.

Radial distribution functions simulated for 50 molecules of HCOOH, corresponding to the adsorption loading of ca. 1 molecule of HCOOH per metal center are compared for Ni-, Co-, Fe-, Mn-, Zn-, and Cu-MOF-74 at 298 K in Figure 8. The presence of three primary binding geometries of HCOOH found in the surroundings of the metal centers is confirmed. The distance between the double bonded O_fa2_ atom and the metal center (configuration I) range from 2.22 Å for Ni-MOF-74 to 2.54 Å for Cu-MOF-74. The binding distances and the corresponding adsorption intensities decrease in M-MOF-74 in the following order: Ni > Co ≈ Fe > Zn > Mn > Cu. In Cu-MOF-74, which has the weakest affinity with HCOOH, configuration III is found to outperform configuration II in terms of stability. Radial distribution functions for the interactions between HCOOH in the M-MOF-74 series are shown in Figure S8 of the Supporting Information. The intensity of hydrogen bond formation increases with decreasing affinity of HCOOH with the framework: Cu-MOF-74 ≈ Zn-MOF-74 > Mn-MOF-74 > Fe-MOF-74 > Co-MOF-74 > Ni-MOF-74. In Zn-, and Cu-MOF-74, the HCOOH dimerization is found to be prevalent over the hydrogen bonds-driven nucleation.

**Figure 9:**
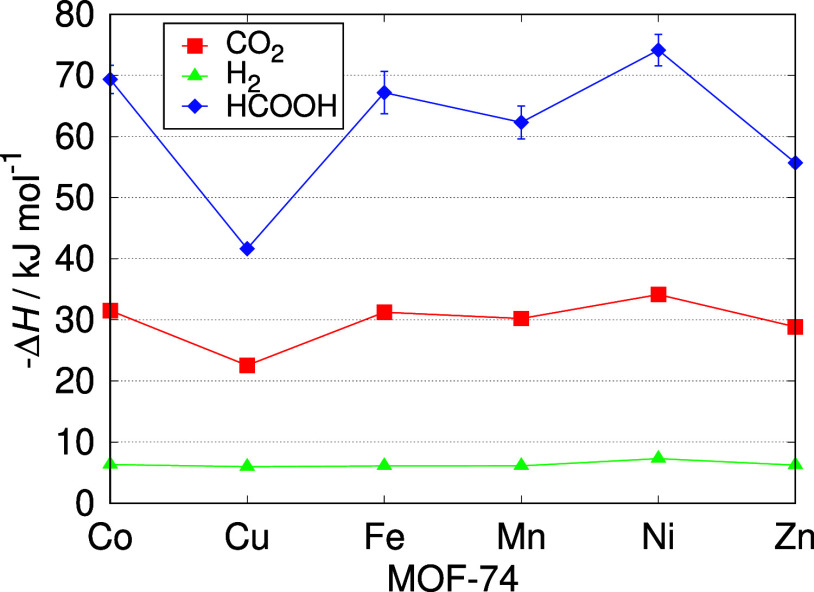
Isosteric heat of adsorption of CO_2_, H_2_ and HCOOH at 298.15 K in M-MOF-74. The isosteric heat of adsorption of HCOOH is significantly higher than the other components of CO_2_ hydrogenation reaction in all the studied frameworks, leading to the selective adsorption of favored HCOOH component. The isosteric heat of adsorption for H_2_ is similar in all the frameworks with a slight advantage for Ni-MOF-74. The lines connecting the symbols are used to guide the eye.

The mole fractions of CO_2_ and H_2_ obtained from the GCMC simulations of the CO_2_ hydrogenation reaction are shown in Figure S9 of the Supporting Information. The mole fractions of adsorbed CO_2_ are significantly higher than the mole fraction of H_2_ at all the studied conditions, showing similarity to the adsorption isotherms of CO_2_/H_2_ mixtures in M-MOF-74, studied by Wasik et al.^38^ The mole fractions of CO_2_ decrease with increasing temperature, except for Ni-, Co-, and Fe-MOF-74 at the temperature range 298.15–350 K, where the mole fraction of CO_2_ slightly increases. The increase is caused by the large decrease in HCOOH production, affecting mole fractions of the other components. The number of adsorbed CO_2_ molecules decreases throughout the range of studied temperatures. The mole fractions of H_2_ increase with temperature, inversely related to the mole fractions of CO_2_ and HCOOH. The highest mole fractions of H_2_ and the lowest mole fractions of CO_2_ and HCOOH are observed for Cu-MOF-74, indicating that HCOOH production is more influenced by the type of metal center than by the quantity of the limiting reagent. The affinity of CO_2_, H_2_ and HCOOH with M-MOF-74 was compared in Figure 9. For all the studied frameworks, the isosteric heat of adsorption of HCOOH was significantly higher (from ca. 74 kJ mol^–1^ in Ni-MOF-74 to ca. 42 kJ mol^–1^ in Cu-MOF-74) than the other components of CO_2_ hydrogenation reaction, followed by CO_2_ (from 34 kJ mol^–1^ in Ni-MOF-74 to 23 kJ mol^–1^ in Cu-MOF-74), and H_2_ (from 7 kJ mol^–1^ in Ni-MOF-74 to 6 kJ mol^–1^ in Cu-MOF-74). This high difference in affinity leads to the selective adsorption of favored HCOOH component, causing its enhanced formation in the CO_2_ hydrogenation reaction. The isosteric heat of adsorption for CO_2_ decreases in the same order as the isosteric heat of adsorption for HCOOH depending on the type of metal center in M-MOF-74: Ni > Co > Fe > Mn > Zn > Cu. The isosteric heat of adsorption for H_2_ is similar in all the frameworks with a slight advantage for Ni-MOF-74.

In Table 1, the HCOOH production from CO_2_ hydrogenation using M-MOF-74 at 298.15 K and 60 bar was compared with previously studied MOF Cu-BTC,^20^ and the most^16^ as well as the least effective^14^ transition metal catalysts. The types of catalysts were selected based on the resulting concentration of the CO_2_ hydrogenation product (*c*_HCOOH_, eq 6). The final concentration of HCOOH obtained from the Ni-MOF-74 confinement (*c*_HCOOH_ = 2.20 mol L^–1^) is only 1.14 times lower than the highest reported concentration obtained with the use of the Wilkinson complex,^16^ and 2200 times higher than the lowest reported concentration obtained using a catalyst with N-heterocyclic carbene ligands.^14^ This is a significant improvement compared to our previous work,^20^ where the application of the best performing MOF Cu-BTC resulted in the concentration of HCOOH ca. 80 times lower than the Wilkinson complex. Notably, the HCOOH concentration obtained using Cu-BTC is ca. 6 times higher than Cu-MOF-74 despite the same type of metal center, reflecting the importance of higher partial charge of the metal cation. Considering the resulting concentration of HCOOH from the confinement of Ni-MOF-74 close to the highest concentration of the formate product obtained with the Wilkinson complex, and a more valuable molecular form of the product, the application of Ni-MOF-74 has the potential to be an economically more attractive method of CO_2_ conversion than transition metal catalysts. The costs of formate downstream processing methods including separation, concentration, and acidification of formate solutions can be avoided.^81^ The cost reduction for the conversion and concentration of 10 wt % formate to 85 wt % formic acid is estimated at $380/ton of FA in an economic analysis performed by Ramdin et al.^81^ Further studies of Ni-MOF-74 toward its ligands functionalization and the analysis of other Ni-based MOFs may be promising research subjects in HCOOH production.

**Table 1:** HCOOH Production from CO_2_ Hydrogenation Using M-MOF-74 at 298.15 K and 60 bar, Compared to Cu-BTC,^20^ and the Most^16^ as well as the Least^14^ Effective Transition Metal Catalysts^a^^14^

catalyst	conditions	product	concentration [mol L^–1^]	references
Wilkinson complex RhCl(PPh_3_)_3_ + 3 PPh_3_	298.15 K, *p*_CO_2__ = 40 bar, *p*_H_2__ = 20 bar, 20 h	HCOOH	2.50	(16)
none	Ni-MOF-74 confinement, 298.15 K, 60 bar	HCOOH	2.20	this work
none	Co-MOF-74 confinement, 298.15 K, 60 bar	HCOOH	0.59	this work
none	Fe-MOF-74 confinement, 298.15 K, 60 bar	HCOOH	0.46	this work
none	Mn-MOF-74 confinement, 298.15 K, 60 bar	HCOOH	0.12	this work
none	Zn-MOF-74 confinement, 298.15 K, 60 bar	HCOOH	0.032	this work
none	Cu-BTC confinement, 298.15 K, 60 bar	HCOOH	0.031	(20)
none	Cu-MOF-74 confinement, 298.15 K, 60 bar	HCOOH	0.005	this work
(η^6^-arene)Ru(bis-NHC) complex no. 1	353.15 K, 40 bar, 1 h	formate	0.001	(14)

a The highest final concentration of HCOOH (*c*_HCOOH_, eq 6) obtained from the Ni-MOF-74 confinement is only 1.14 times lower than the highest reported concentration obtained with the use of the Wilkinson complex,^16^ and 2200 times higher than the lowest reported concentration obtained using a catalyst with N-heterocyclic carbene ligands.^14^

## 4 Conclusions

We carried out Monte Carlo and DFT simulations to study the performance of M-MOF-74, where M = Ni, Cu, Co, Fe, Mn, Zn, for the adsorption and production process of formic acid. The nonpolarizable CO_2_, and H_2_ force field for adsorption in M-MOF-74^38^ was evaluated for compatibility with a variant of the OPLS/AA force field for HCOOH.^42^ The loading and heat of HCOOH adsorption were found to depend on the metal center as follows: Ni > Co > Fe > Mn > Zn > Cu, which suggests that HCOOH adsorption is predominantly influenced by the electrostatic interactions dependent on the effective charge of the M^2+^ ion at the open-metal site. The two-step mechanism of adsorption, wherein HCOOH molecules preferably adsorb at the metal centers, followed by adsorption above a triangle of oxygen atoms within the framework, was present in the adsorption isotherms of Ni-, Co-, Fe-, and Mn-MOF-74 frameworks. The three primary binding geometries of HCOOH adsorption in M-MOF-4 were found in the surroundings of the metal centers using Baker’s minimization method. The binding geometries and energies of the most stable configuration computed from force field-based simulations agree with DFT calculations. The effect of the type of metal centers on the yield of HCOOH from the CO_2_ hydrogenation reaction carried out in confinement was analyzed in M-MOF-74, and compared with the literature data for Cu-BTC and transition metal catalysts. The adsorption isobars of the studied systems were computed with Monte Carlo simulations in the grand-canonical ensemble, and the enhancement in HCOOH production was calculated. The performance of M-MOF-74 in the production of HCOOH in confinement was shown to follow the same order as the uptake and the heat of HCOOH adsorption. The application of the Ni-MOF-74 framework results in the highest enhancement in HCOOH production. The obtained mole fraction of HCOOH equals ca. 0.1 at 298.15 K, 60 bar, which is ca. 10^5^ times higher compared to the gas phase. The final concentration of HCOOH resulting from the Ni-MOF-74 confinement (*c*_HCOOH_ = 2.20 mol L^–1^) is only 1.14 times lower than the highest reported concentration obtained with the use of the Wilkinson complex, and 2200 times higher than the lowest reported concentration obtained using a catalyst with N-heterocyclic carbene ligands. This is a major improvement compared to our previous work, where the application of the best performing MOF Cu-BTC resulted in ca. 2000 times higher HCOOH mole fraction compared to the gas phase, and the final concentration of HCOOH ca. 80 times lower than obtained with the Wilkinson complex. The metal–organic framework Ni-MOF-74 has comparable performance to the most effective transition metal catalyst and an additional advantage of a more valuable molecular form of the product. An economic and carbon emission analysis should be carried out to fully investigate the potential of Ni-MOF-74 as a useful alternative to transition metal catalysts. An interesting topic for future research is the study of ligands functionalization and the review of other Ni-based MOFs that may be promising in the HCOOH production from the CO_2_ hydrogenation reaction.

## Supporting Information Available

Lennard–Jones and Coulombic interactions for CO_2_, H_2_, and HCOOH; schematic representation of M-MOF-74 (M = Ni, Cu, Co, Fe, Mn, Zn) structures and the HCOOH model for DFT; lattice parameters for M-MOF-74 (M = Ni, Cu, Co, Fe, Mn, Zn) unit cells resulting from the geometry optimization from DFT; initial gas-phase mole fractions of CO_2_, H_2_, and HCOOH used as an input for grand-canonical Monte Carlo simulations at 298.15–800 K, and 1–60 bar; distribution of the HCOOH molecules inside M-MOF-74 (M = Co, Fe, Mn, Zn) analyzed using density profiles from grand-canonical Monte Carlo simulations at 298 K, 10 kPa; binding energies of the most stable configuration of HCOOH in M-MOF-74 (M = Ni, Cu, Co, Fe, Mn, Zn) obtained from force field-based molecular simulations and DFT; mole fractions of CO_2_, H_2_, and HCOOH obtained from grand-canonical Monte Carlo simulations in M-MOF-74 (M = Ni, Cu, Co, Fe, Mn, Zn) at 298.15–800 K, and 1–60 bar; radial distribution functions for HCOOH in M-MOF-74 (M = Ni, Cu, Co, Fe, Mn, Zn) (PDF)
