# Supplementary material for: The Impact of Metal Centers in the M-MOF-74 Series on Formic Acid Production
Source: ACS Appl Mater Interfaces. 2024 Aug 14;16(34):45006–19. doi: 10.1021/acsami.4c10678 (PMC11367578; doi:10.1021/acsami.4c10678)
Supplement: Supplementary file 1 — am4c10678_si_001.pdf [file am4c10678_si_001.pdf]

Supporting Information: The Impact of Metal Centers in the M-MOF-74 Series on Formic Acid Production

Dominika O. Wasik,[†,‡] José Manuel Vicent-Luna,[*,†] Shima Rezaie,[¶] Azahara Luna-Triguero,[¶,‡] Thijs J. H. Vlugt,[§] and Sofía Calero[*,†,‡]

†*Materials Simulation and Modelling, Department of Applied Physics, Eindhoven University of Technology, 5600MB Eindhoven, The Netherlands*

‡*Eindhoven Institute for Renewable Energy Systems, Eindhoven University of Technology, PO Box 513, Eindhoven 5600 MB, The Netherlands*

¶*Energy Technology, Department of Mechanical Engineering, Eindhoven University of Technology, 5600MB Eindhoven, The Netherlands*

§*Engineering Thermodynamics, Process & Energy Department, Faculty of Mechanical, Maritime and Materials Engineering, Delft University of Technology, Leeghwaterstraat 39, Delft 2628CB, The Netherlands*

E-mail: j.vicent.luna@tue.nl; s.calero@tue.nl

- Lennard-Jones and Coulombic interactions for CO_2, H_2, and HCOOH;
- Schematic representation of M-MOF-74 (M = Ni, Cu, Co, Fe, Mn, Zn) structures and the HCOOH model for DFT;
- Lattice parameters for M-MOF-74 (M = Ni, Cu, Co, Fe, Mn, Zn) unit cells resulting from the geometry optimization from DFT;
- Initial gas-phase mole fractions of CO_2, H_2, and HCOOH used as an input for grand-canonical Monte Carlo simulations at 298.15 - 800 K, and 1 - 60 bar;
- Distribution of the HCOOH molecules inside M-MOF-74 (M = Co, Fe, Mn, Zn) analyzed using density profiles from grand-canonical Monte Carlo simulations at 298 K, 10 kPa;
- Binding energies of the most stable configuration of HCOOH in M-MOF-74 (M = Ni, Cu, Co, Fe, Mn, Zn) obtained from force field-based molecular simulations and DFT;
- Mole fractions of CO_2, H_2, and HCOOH obtained from grand-canonical Monte Carlo simulations in M-MOF-74 (M = Ni, Cu, Co, Fe, Mn, Zn) at 298.15 - 800 K, and 1 - 60 bar;
- Radial distribution functions for HCOOH in M-MOF-74 (M = Ni, Cu, Co, Fe, Mn, Zn).

Table S1: Lennard-Jones and Coulombic interaction potentials for CO_2,[1,2] H_2,[3] and HCOOH,[4] with an exception/override to the use of the Lorentz-Berthelot mixing rules[5] for H_{com}–Me interactions from the study of Wasik et al.[6] For Lennard-Jones and Coulombic interaction potentials for the M-MOF-74 (M = Ni, Cu, Co, Fe, Mn, Zn) frameworks, the reader is referred to the study of Wasik et al.[6] All molecules are charge-neutral. The schematic representation of the HCOOH molecule with the atoms labelled is visualized in Figure 4c of the main text.

Atom	ϵ/k_B /[K]	σ /[Å]	q /[e$^-$]
O_{CO_2}	85.671	3.017	-0.3256
C_{CO_2}	29.93	2.742	0.6512
H_{com}	36.7	2.958	-0.936
H_{H_2}	0	0	0.468
C_{fa1}	49.6728	3.67	0.52
O_{fa2}	99.34559	2.9	-0.44
H_{fa1}	7.09611	2.37	0
O_{fa1}	80.46271	2.94	-0.53
H_{fa2}	1	1	0.45
Lennard-Jones interactions overrides			
H_{com} - Ni	216.4823	2.1932	
H_{com} - Cu	11.5357	3.036	
H_{com} - Co	17.6963	2.2068	
H_{com} - Fe	18.5939	2.776	
H_{com} - Mn	13.9529	2.798	
H_{com} - Zn	43.069	2.7095	

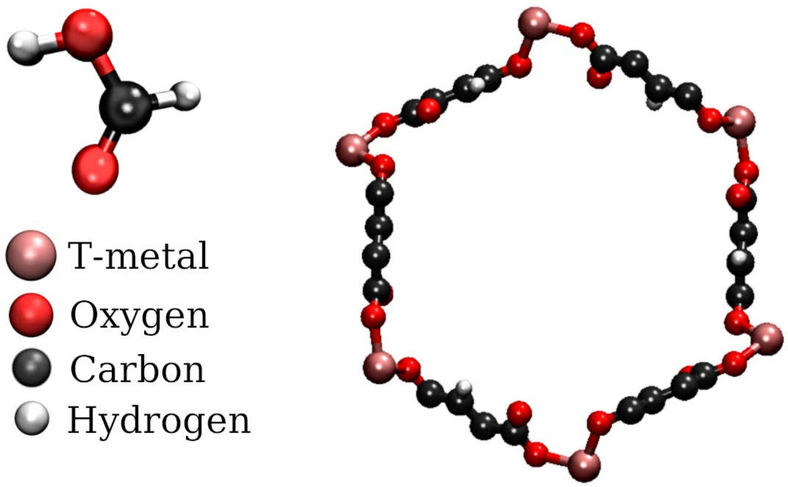

Figure S1. Schematic representation of: (a) a HCOOH molecule, and (b) M-MOF-74 unit cell (T-Metals: Co, Cu, Fe, Mn, Ni, and Zn), used in Density Functional Theory calculations.

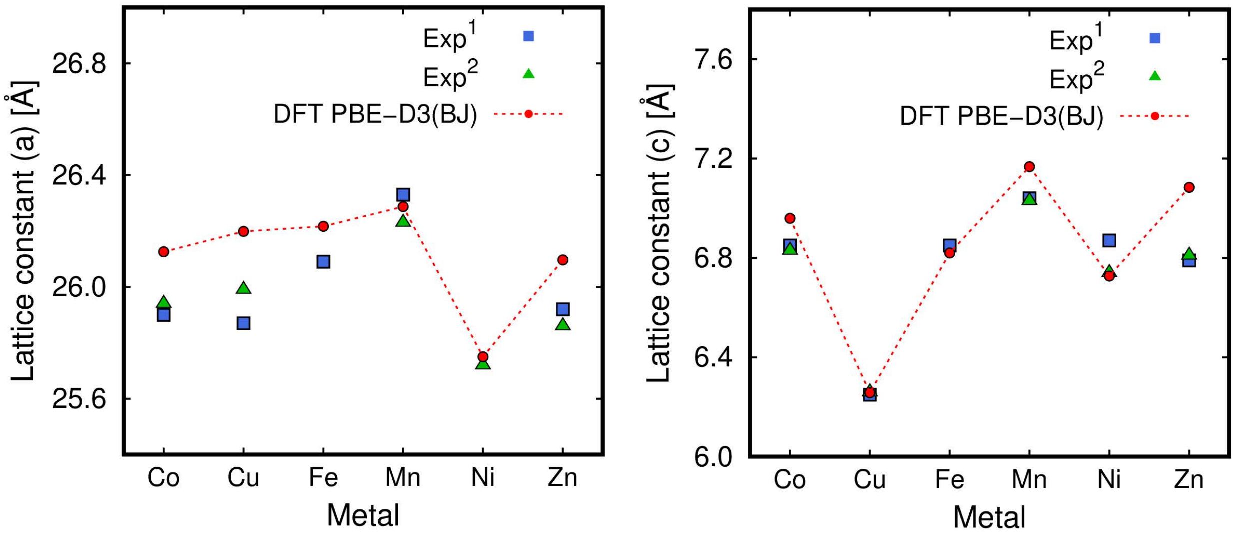

Figure S2. Lattice parameters resulting from the geometry optimization calculated from Density Functional Theory using the M-MOF-74 unit cell (M = Co, Cu, Fe, Mn, Ni, and Zn) shown in Figure S2 of the Supporting Information. The calculated values show a strong concordance with the literature data.[7,8]

Table S2: Initial gas-phase mole fractions of CO_2, H_2, and HCOOH obtained in the study of Wasik et al.[9] from Continuous Fractional Component Monte Carlo simulations[10–12] in the Reaction Ensemble[13–15] at 298.15 - 800 K and 1 - 60 bar. The mole fractions computed from the Rx/CFC simulations are used as an input for the GCMC ensemble. The subscripts show uncertainties computed using error propagation rules. Reproduced from ref.[9] Available under a CC-BY 4.0 license. Copyright © 2023 The Author(s). Published by Elsevier B.V.

P/[bar]	T/[K]	x_{CO_2,H_2}	x_{HCOOH} / 10^{-6}
1	298.15	$0.49999999_{1\cdot10^{-8}}$	$0.017_{0.008}$
	400	$0.49999998_{2\cdot10^{-8}}$	$0.04_{0.01}$
	500	$0.49999995_{2\cdot10^{-8}}$	$0.10_{0.02}$
	600	$0.49999993_{3\cdot10^{-8}}$	$0.14_{0.02}$
	700	$0.49999991_{4\cdot10^{-8}}$	$0.17_{0.03}$
	800	$0.49999991_{4\cdot10^{-8}}$	$0.18_{0.03}$
5	298.15	$0.49999996_{4\cdot10^{-8}}$	$0.08_{0.03}$
	400	$0.49999988_{3\cdot10^{-8}}$	$0.24_{0.02}$
	500	$0.49999979_{9\cdot10^{-8}}$	$0.42_{0.07}$
	600	$0.49999966_{7\cdot10^{-8}}$	$0.68_{0.05}$
	700	$0.4999996_{1\cdot10^{-7}}$	$0.8_{0.1}$
	800	$0.49999950_{9\cdot10^{-8}}$	$1.00_{0.07}$
10	298.15	$0.49999994_{3\cdot10^{-8}}$	$0.13_{0.03}$
	400	$0.49999977_{3\cdot10^{-8}}$	$0.46_{0.02}$
	500	$0.49999956_{4\cdot10^{-8}}$	$0.88_{0.03}$
	600	$0.49999935_{8\cdot10^{-8}}$	$1.30_{0.06}$
	700	$0.4999992_{1\cdot10^{-7}}$	$1.6_{0.1}$
	800	$0.4999990_{1\cdot10^{-7}}$	$1.92_{0.09}$
15	298.15	$0.49999988_{4\cdot10^{-8}}$	$0.24_{0.03}$
	400	$0.49999967_{6\cdot10^{-8}}$	$0.66_{0.05}$
	500	$0.4999994_{1\cdot10^{-7}}$	$1.3_{0.1}$
	600	$0.4999991_{2\cdot10^{-7}}$	$1.9_{0.1}$
	700	$0.4999988_{1\cdot10^{-7}}$	$2.4_{0.1}$
	800	$0.4999986_{2\cdot10^{-7}}$	$2.7_{0.1}$
20	298.15	$0.49999987_{4\cdot10^{-8}}$	$0.25_{0.03}$
	400	$0.49999952_{3\cdot10^{-8}}$	$0.96_{0.03}$
	500	$0.49999916_{7\cdot10^{-8}}$	$1.68_{0.06}$
	600	$0.4999988_{2\cdot10^{-7}}$	$2.5_{0.1}$

	700	$0.4999984_{1\cdot10^{-7}}$	$3.2_{0.1}$
	800	$0.4999981_{3\cdot10^{-7}}$	$3.8_{0.2}$
25	298.15	$0.49999983_{7\cdot10^{-8}}$	$0.35_{0.05}$
	400	$0.4999995_{1\cdot10^{-7}}$	$1.0_{0.1}$
	500	$0.4999989_{2\cdot10^{-7}}$	$2.1_{0.1}$
	600	$0.4999984_{2\cdot10^{-7}}$	$3.1_{0.1}$
	700	$0.4999980_{1\cdot10^{-7}}$	$3.93_{0.09}$
	800	$0.4999977_{1\cdot10^{-7}}$	$4.7_{0.1}$
30	298.15	$0.49999984_{5\cdot10^{-8}}$	$0.33_{0.04}$
	400	$0.4999993_{1\cdot10^{-7}}$	$1.38_{0.08}$
	500	$0.4999987_{2\cdot10^{-7}}$	$2.5_{0.1}$
	600	$0.4999981_{2\cdot10^{-7}}$	$3.8_{0.2}$
	700	$0.4999976_{2\cdot10^{-7}}$	$4.8_{0.2}$
	800	$0.4999973_{2\cdot10^{-7}}$	$5.5_{0.2}$
40	298.15	$0.49999972_{8\cdot10^{-8}}$	$0.56_{0.06}$
	350	$0.4999995_{2\cdot10^{-7}}$	$1.1_{0.1}$
	400	$0.49999909_{8\cdot10^{-8}}$	$1.82_{0.06}$
	500	$0.4999983_{2\cdot10^{-7}}$	$3.4_{0.2}$
	600	$0.4999976_{2\cdot10^{-7}}$	$4.7_{0.1}$
	700	$0.4999969_{2\cdot10^{-7}}$	$6.2_{0.2}$
	800	$0.4999964_{3\cdot10^{-7}}$	$7.2_{0.2}$
50	298.15	$0.4999996_{3\cdot10^{-7}}$	$0.8_{0.2}$
	350	$0.4999993_{2\cdot10^{-7}}$	$1.3_{0.2}$
	400	$0.4999989_{1\cdot10^{-7}}$	$2.10_{0.09}$
	500	$0.4999980_{1\cdot10^{-7}}$	$4.1_{0.1}$
	600	$0.4999970_{3\cdot10^{-7}}$	$6.0_{0.2}$
	700	$0.4999962_{3\cdot10^{-7}}$	$7.6_{0.2}$
	800	$0.4999954_{3\cdot10^{-7}}$	$9.1_{0.3}$
60	298.15	$0.4999996_{1\cdot10^{-7}}$	$0.9_{0.1}$
	350	$0.49999920_{9\cdot10^{-8}}$	$1.61_{0.07}$
	400	$0.4999987_{2\cdot10^{-7}}$	$2.6_{0.1}$
	500	$0.4999975_{2\cdot10^{-7}}$	$4.9_{0.2}$
	600	$0.49999650_{7\cdot10^{-8}}$	$7.01_{0.06}$
	700	$0.4999954_{3\cdot10^{-7}}$	$9.3_{0.2}$
	800	$0.4999946_{3\cdot10^{-7}}$	$10.9_{0.2}$

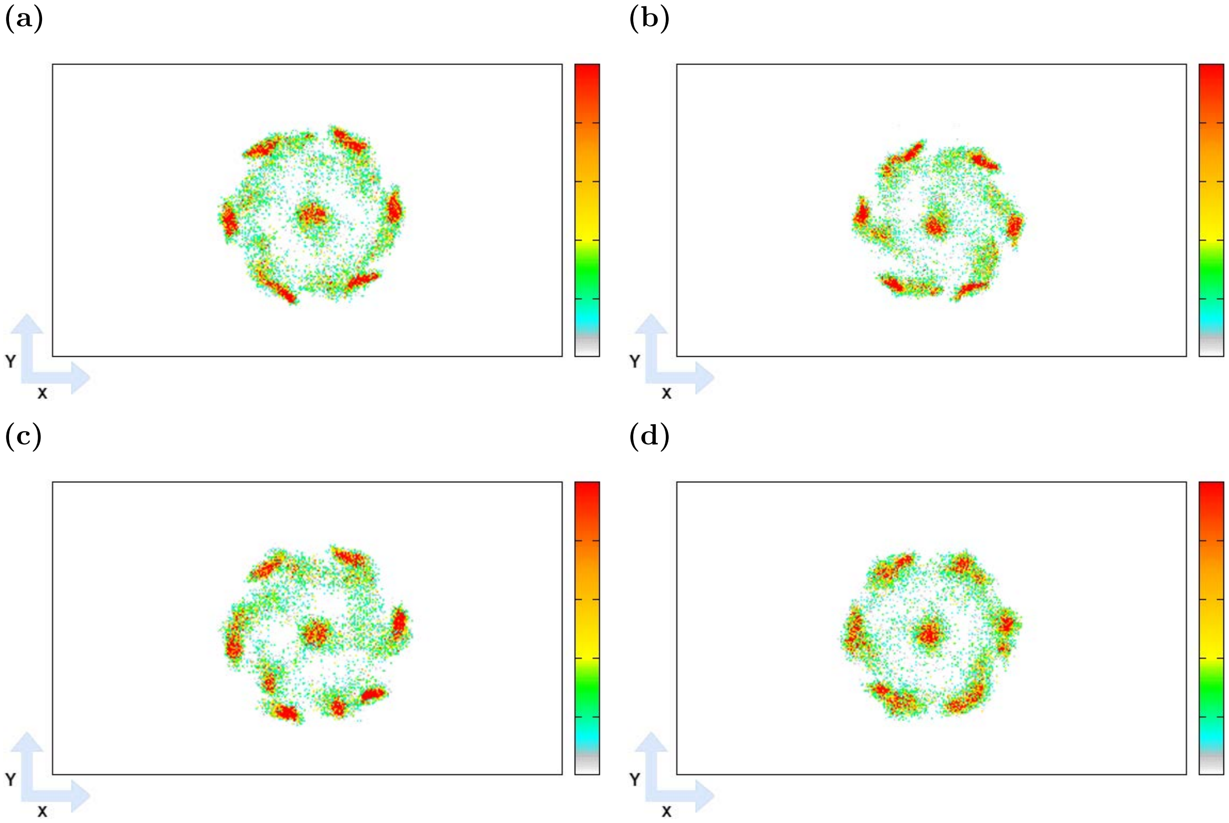

Figure S3. Distribution of the HCOOH molecules analyzed using density profiles from GCMC simulations at 298 K, 10 kPa in: (a) Co-MOF-74, (b) Fe-MOF-74, (c) Mn-MOF-74, (d) Zn-MOF-74. The center of mass of the molecules that are adsorbed was projected onto the XY plane. The color gradation of the scales relates to the most and least populated regions of the structure, which is relative in each case. The color scale is shown as a reference for the loading. The preferential sites of HCOOH (colored red) in all structures are at the open-metal centers.

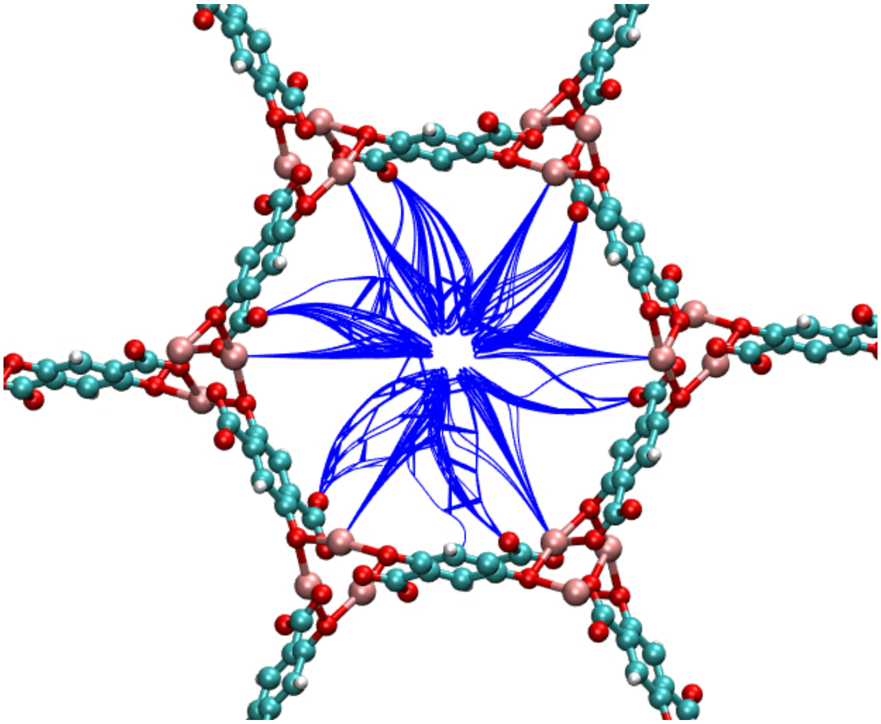

Figure S4. Electrostatic field lines within the cavities of M-MOF-74 (M = Co, Cu, Fe, Mn, Ni, and Zn) from the positively charged metal sites to the electronegative Oa atoms. For details about calculation the reader is referred to the study by Luna-Triguero et al.[16] Reproduced from ref.[16] Copyright © 2019 American Chemical Society.

Table S3: Normalized Coulombic charges for M-MOF-74 (M = Ni, Cu, Co, Fe, Mn, Zn) with respect to the partial charge of the metal center. The charges used for analysis are from the study of Wasik et al.[6] The calculated relative charges show high similarity for each type of atoms identified in all the frameworks. The subscripts show the deviation in the average partial charges of the atom types through the M-MOF-74 series.

Atom	Co	Cu	Fe	Mn	Ni	Zn	Average normalized charge
Me	1	1	1	1	1	1	1
Ca	0.363	0.414	0.290	0.317	0.389	0.341	$0.35_{0.04}$
Cb	-0.155	-0.167	-0.121	-0.151	-0.177	-0.154	$-0.15_{0.02}$
Cc	-0.094	-0.091	-0.072	-0.055	-0.055	-0.083	$-0.07_{0.02}$
Cd	0.184	0.187	0.143	0.151	0.177	0.160	$0.17_{0.02}$
Oa	-0.407	-0.441	-0.358	-0.382	-0.406	-0.391	$-0.40_{0.03}$
Ob	-0.504	-0.517	-0.491	-0.472	-0.526	-0.476	$-0.50_{0.02}$
Oc	-0.458	-0.472	-0.458	-0.450	-0.453	-0.461	$-0.459_{0.007}$
H	0.071	0.088	0.066	0.041	0.052	0.064	$0.06_{0.01}$

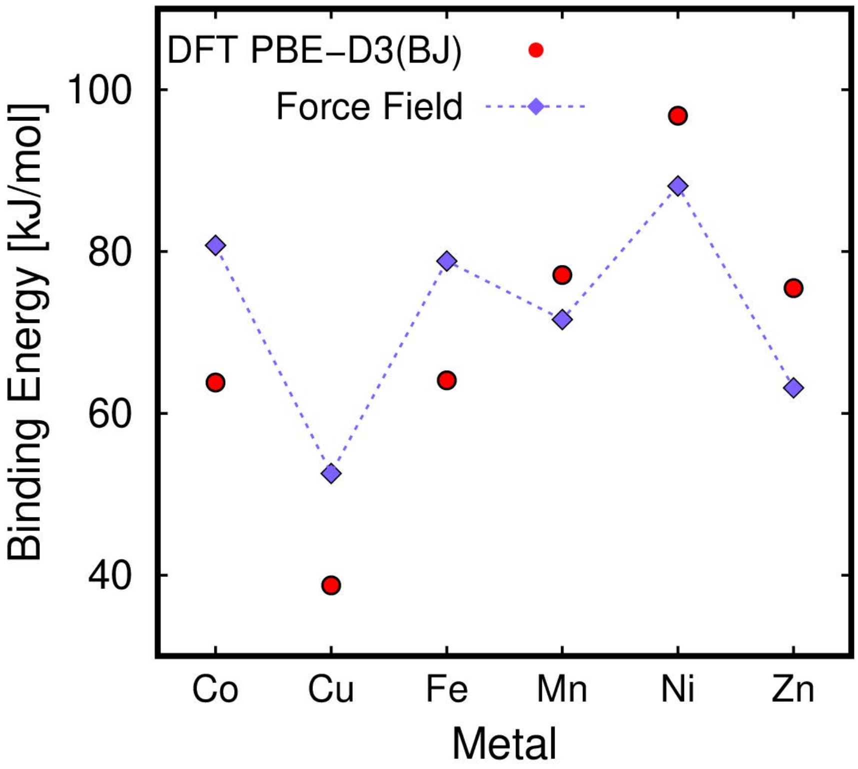

Figure S5. Absolute values of binding energies of the most stable configuration of HCOOH in M-MOF-74 (M = Co, Cu, Fe, Mn, Ni, and Zn) obtained from force field-based molecular simulations compared to the DFT (PBE-D3(BJ)) results. The binding energies vary between the selected methods but follow a similar general trend across the different metals. Both computational methods indicate that Cu-MOF-74 has the weakest binding affinity, while Ni-MOF-74 shows the strongest binding affinity. The binding energy in Cu-MOF-74 is ca. 40 kJ mol^{-1} for DFT, while it is significantly higher (ca. 50 kJ mol^{-1}) for force field simulations. The force field-based simulations show a binding energy of HCOOH in Ni-MOF-74 ca. 90 kJ mol^{-1}, whereas DFT shows a higher value, close to ca. 100 kJ mol^{-1}. The relative difference between the two methods is 18%. The lines connecting the symbols are used to guide the eye.

Table S4: Mole fractions of CO_2, H_2, and HCOOH obtained using Monte Carlo simulations in the grand-canonical ensemble in the Ni-MOF-74 framework at 298.15 - 800 K and 1 - 60 bar. The mole fractions of CO_2, H_2, and HCOOH at reaction equilibrium obtained from the Continuous Fractional Component Monte Carlo simulations in the Reaction Ensemble[9] were used in the GCMC simulations as input. The subscripts show uncertainties computed using error propagation rules.

P/[bar]	T/[K]	x_{CO_2}	x_{H_2}	x_{HCOOH} / 10^{-6}
1	298.15	$0.988_{0.008}$	$0.0017_{0.0001}$	$9875.5_{1088.1}$
	400	$0.981_{0.190}$	$0.019_{0.004}$	$78.8_{93.3}$
	500	$0.928_{0.058}$	$0.072_{0.005}$	$3.1_{5.7}$
	600	$0.852_{0.038}$	$0.148_{0.012}$	$1.7_{3.1}$
	700	$0.791_{0.020}$	$0.209_{0.005}$	$4.1_{11.3}$
	800	$0.740_{0.031}$	$0.260_{0.011}$	0_{0}
5	298.15	$0.964_{0.014}$	$0.0031_{0.0002}$	$32487.4_{6584.2}$
	400	$0.979_{0.018}$	$0.019_{0.001}$	$1288.7_{374.2}$
	500	$0.925_{0.039}$	$0.075_{0.004}$	$123.0_{81.7}$
	600	$0.855_{0.021}$	$0.145_{0.003}$	$39.9_{35.3}$
	700	$0.792_{0.021}$	$0.208_{0.004}$	$12.2_{16.5}$
	800	$0.737_{0.012}$	$0.263_{0.005}$	0_{0}
10	298.15	$0.958_{0.011}$	$0.0033_{0.0002}$	$38650.0_{5439.5}$
	400	$0.979_{0.016}$	$0.0195_{0.0005}$	$1984.1_{833.2}$
	500	$0.925_{0.033}$	$0.074_{0.002}$	$399.5_{185.4}$
	600	$0.856_{0.024}$	$0.144_{0.005}$	$87.5_{107.1}$
	700	$0.790_{0.007}$	$0.210_{0.003}$	$32.4_{24.1}$
	800	$0.738_{0.005}$	$0.262_{0.003}$	$12.8_{9.8}$
15	298.15	$0.931_{0.011}$	$0.004_{0.001}$	$64600.9_{5715.0}$
	400	$0.977_{0.007}$	$0.020_{0.001}$	$3191.7_{628.1}$
	500	$0.926_{0.018}$	$0.074_{0.002}$	$360.0_{103.4}$
	600	$0.856_{0.008}$	$0.144_{0.002}$	$129.9_{40.6}$
	700	$0.789_{0.014}$	$0.210_{0.004}$	$23.4_{24.5}$
	800	$0.738_{0.006}$	$0.262_{0.002}$	$21.2_{20.0}$
20	298.15	$0.941_{0.019}$	$0.0040_{0.0002}$	$54877.4_{10573.5}$
	400	$0.975_{0.007}$	$0.0200_{0.0004}$	$4678.6_{382.8}$
	500	$0.925_{0.023}$	$0.075_{0.002}$	$460.3_{283.5}$
	600	$0.857_{0.017}$	$0.143_{0.002}$	$139.5_{56.1}$

	700	0.790 0.011	0.210 0.002	48.4 19.1
	800	0.738 0.007	0.262 0.003	33.2 26.1
25	298.15	0.925 0.009	0.0042 0.0004	71231.6 4718.0
	400	0.975 0.006	0.0207 0.0003	3926.0 702.6
	500	0.926 0.024	0.073 0.002	524.5 55.6
	600	0.855 0.014	0.145 0.002	201.9 73.3
	700	0.791 0.004	0.209 0.002	63.0 23.3
	800	0.739 0.006	0.261 0.003	30.0 9.2
30	298.15	0.934 0.014	0.0048 0.0005	61063.8 10092.7
	400	0.973 0.007	0.022 0.001	5543.2 579.7
	500	0.927 0.017	0.072 0.002	831.1 178.7
	600	0.855 0.019	0.144 0.003	170.1 39.2
	700	0.790 0.011	0.210 0.003	67.2 29.8
	800	0.738 0.007	0.262 0.003	54.4 15.2
40	298.15	0.899 0.025	0.0048 0.0003	95858.1 13522.2
	350	0.968 0.007	0.012 0.001	20183.1 846.6
	400	0.970 0.005	0.023 0.001	6950.5 765.7
	500	0.926 0.010	0.073 0.002	1158.7 319.0
	600	0.856 0.015	0.143 0.003	246.1 105.2
	700	0.791 0.009	0.209 0.002	101.7 21.6
	800	0.736 0.006	0.264 0.003	54.2 11.0
50	298.15	0.879 0.028	0.0053 0.0003	115594.3 16340.3
	350	0.963 0.008	0.013 0.001	23487.9 3197.9
	400	0.968 0.005	0.024 0.001	7704.2 600.9
	500	0.926 0.009	0.073 0.001	1254.6 186.0
	600	0.854 0.010	0.146 0.002	315.3 125.0
	700	0.789 0.013	0.210 0.003	112.9 31.2
	800	0.737 0.003	0.263 0.002	71.5 23.4
60	298.15	0.889 0.034	0.006 0.001	104742.3 18172.0
	350	0.960 0.002	0.0141 0.0002	25871.3 775.2
	400	0.965 0.004	0.025 0.002	9561.3 1045.5
	500	0.925 0.008	0.074 0.003	1663.0 231.5
	600	0.856 0.012	0.144 0.002	367.4 73.0
	700	0.790 0.009	0.210 0.003	161.3 59.6
	800	0.736 0.005	0.264 0.002	92.2 15.2

Table S5: Mole fractions of CO_2, H_2, and HCOOH obtained using Monte Carlo simulations in the grand-canonical ensemble in the Cu-MOF-74 framework at 298.15 - 800 K and 1 - 60 bar. The mole fractions of CO_2, H_2, and HCOOH at reaction equilibrium obtained from the Continuous Fractional Component Monte Carlo simulations in the Reaction Ensemble[9] were used in the GCMC simulations as input. The subscripts show uncertainties computed using error propagation rules.

P/[bar]	T/[K]	x_{CO_2}	x_{H_2}	x_{HCOOH} / 10^{-6}
1	298.15	$0.990_{0.018}$	$0.0100_{0.0003}$	$20.0_{36.9}$
	400	$0.943_{0.038}$	$0.057_{0.005}$	0_{0}
	500	$0.872_{0.013}$	$0.128_{0.007}$	0_{0}
	600	$0.812_{0.024}$	$0.188_{0.006}$	0_{0}
	700	$0.755_{0.011}$	$0.245_{0.009}$	$9.6_{26.6}$
	800	$0.712_{0.018}$	$0.288_{0.007}$	0_{0}
5	298.15	$0.993_{0.004}$	$0.0065_{0.0004}$	$37.8_{30.2}$
	400	$0.942_{0.016}$	$0.058_{0.001}$	$30.2_{59.1}$
	500	$0.874_{0.006}$	$0.126_{0.003}$	$0.8_{1.3}$
	600	$0.808_{0.008}$	$0.192_{0.005}$	$4.4_{5.4}$
	700	$0.758_{0.009}$	$0.242_{0.003}$	0_{0}
	800	$0.713_{0.008}$	$0.287_{0.003}$	0_{0}
10	298.15	$0.994_{0.007}$	$0.0058_{0.0005}$	$77.8_{48.2}$
	400	$0.945_{0.011}$	$0.0549_{0.0007}$	$18.7_{16.3}$
	500	$0.876_{0.008}$	$0.124_{0.002}$	$5.7_{6.5}$
	600	$0.811_{0.004}$	$0.189_{0.001}$	$5.1_{8.7}$
	700	$0.755_{0.004}$	$0.245_{0.004}$	$5.8_{7.0}$
	800	$0.713_{0.007}$	$0.287_{0.003}$	$7.7_{14.3}$
15	298.15	$0.994_{0.008}$	$0.0059_{0.0007}$	$74.3_{59.8}$
	400	$0.948_{0.002}$	$0.052_{0.001}$	$19.7_{20.3}$
	500	$0.876_{0.006}$	$0.124_{0.002}$	$12.1_{14.0}$
	600	$0.811_{0.004}$	$0.189_{0.002}$	$4.7_{5.8}$
	700	$0.756_{0.008}$	$0.244_{0.002}$	$14.5_{14.3}$
	800	$0.712_{0.008}$	$0.288_{0.004}$	$5.4_{6.7}$
20	298.15	$0.994_{0.006}$	$0.0059_{0.0003}$	$101.7_{23.9}$
	400	$0.949_{0.007}$	$0.0508_{0.0007}$	$33.6_{19.2}$
	500	$0.876_{0.006}$	$0.124_{0.001}$	$16.6_{11.8}$
	600	$0.810_{0.009}$	$0.190_{0.002}$	$16.1_{12.1}$

	700	$0.756_{0.003}$	$0.2443_{0.0008}$	$7.2_{3.9}$
	800	$0.712_{0.005}$	$0.288_{0.003}$	$5.8_{7.6}$
25	298.15	$0.994_{0.004}$	$0.0061_{0.0005}$	$181.4_{54.6}$
	400	$0.951_{0.011}$	$0.0489_{0.0006}$	$29.0_{15.3}$
	500	$0.877_{0.009}$	$0.123_{0.001}$	$16.2_{8.9}$
	600	$0.811_{0.004}$	$0.189_{0.001}$	$10.8_{6.4}$
	700	$0.756_{0.008}$	$0.244_{0.002}$	$16.5_{12.1}$
	800	$0.712_{0.004}$	$0.288_{0.002}$	$15.5_{19.1}$
30	298.15	$0.993_{0.003}$	$0.0066_{0.0002}$	$149.7_{70.6}$
	400	$0.951_{0.006}$	$0.0487_{0.0003}$	$40.6_{9.1}$
	500	$0.878_{0.002}$	$0.1223_{0.0009}$	$26.6_{12.6}$
	600	$0.811_{0.002}$	$0.189_{0.001}$	$18.2_{10.3}$
	700	$0.755_{0.007}$	$0.245_{0.003}$	$21.3_{11.0}$
	800	$0.711_{0.003}$	$0.289_{0.003}$	$18.6_{21.1}$
40	298.15	$0.993_{0.009}$	$0.007_{0.002}$	$255.5_{188.6}$
	350	$0.979_{0.003}$	$0.0212_{0.0002}$	$94.9_{36.1}$
	400	$0.953_{0.008}$	$0.0473_{0.0008}$	$65.7_{22.7}$
	500	$0.878_{0.006}$	$0.1220_{0.0007}$	$29.9_{6.4}$
	600	$0.810_{0.004}$	$0.190_{0.002}$	$30.8_{9.0}$
	700	$0.755_{0.005}$	$0.245_{0.002}$	$16.0_{6.2}$
	800	$0.711_{0.005}$	$0.289_{0.002}$	$17.8_{3.3}$
50	298.15	$0.992_{0.003}$	$0.0077_{0.0004}$	$275.3_{66.3}$
	350	$0.978_{0.004}$	$0.0221_{0.0003}$	$106.2_{59.5}$
	400	$0.953_{0.003}$	$0.0474_{0.0006}$	$64.0_{17.5}$
	500	$0.879_{0.003}$	$0.121_{0.002}$	$39.6_{18.1}$
	600	$0.810_{0.003}$	$0.190_{0.001}$	$41.0_{19.7}$
	700	$0.755_{0.006}$	$0.245_{0.002}$	$28.3_{10.1}$
	800	$0.711_{0.005}$	$0.289_{0.002}$	$19.8_{9.8}$
60	298.15	$0.992_{0.005}$	$0.0082_{0.0006}$	$244.1_{166.6}$
	350	$0.977_{0.005}$	$0.0225_{0.0006}$	$138.1_{38.6}$
	400	$0.952_{0.005}$	$0.0479_{0.0003}$	$90.8_{34.7}$
	500	$0.879_{0.004}$	$0.121_{0.001}$	$42.2_{10.6}$
	600	$0.810_{0.005}$	$0.190_{0.001}$	$39.2_{15.6}$
	700	$0.754_{0.006}$	$0.246_{0.002}$	$32.9_{10.2}$
	800	$0.710_{0.004}$	$0.290_{0.001}$	$30.6_{10.6}$

Table S6: Mole fractions of CO_2, H_2, and HCOOH obtained using Monte Carlo simulations in the grand-canonical ensemble in the Co-MOF-74 framework at 298.15 - 800 K and 1 - 60 bar. The mole fractions of CO_2, H_2, and HCOOH at reaction equilibrium obtained from the Continuous Fractional Component Monte Carlo simulations in the Reaction Ensemble[9] were used in the GCMC simulations as input. The subscripts show uncertainties computed using error propagation rules.

P/[bar]	T/[K]	x_{CO_2}	x_{H_2}	x_{HCOOH} / 10^{-6}
1	298.15	$0.996_{0.010}$	$0.0021_{0.0001}$	$2365.8_{545.3}$
	400	$0.974_{0.059}$	$0.026_{0.002}$	$41.6_{53.2}$
	500	$0.922_{0.037}$	$0.078_{0.004}$	$18.5_{50.3}$
	600	$0.848_{0.028}$	$0.152_{0.007}$	$59.0_{163.8}$
	700	$0.790_{0.021}$	$0.210_{0.010}$	0_{0}
	800	$0.736_{0.021}$	$0.264_{0.006}$	0_{0}
5	298.15	$0.990_{0.006}$	$0.0032_{0.0001}$	$6848.9_{723.5}$
	400	$0.975_{0.047}$	$0.024_{0.003}$	$369.2_{317.2}$
	500	$0.919_{0.036}$	$0.081_{0.004}$	$61.6_{135.7}$
	600	$0.849_{0.019}$	$0.151_{0.005}$	$9.1_{10.3}$
	700	$0.790_{0.009}$	$0.210_{0.003}$	$5.3_{10.1}$
	800	$0.737_{0.006}$	$0.263_{0.004}$	$8.4_{10.5}$
10	298.15	$0.988_{0.004}$	$0.0035_{0.0003}$	$8536.9_{922.3}$
	400	$0.976_{0.022}$	$0.024_{0.001}$	$764.0_{462.3}$
	500	$0.919_{0.023}$	$0.081_{0.004}$	$120.4_{84.6}$
	600	$0.851_{0.011}$	$0.149_{0.001}$	$35.1_{23.1}$
	700	$0.789_{0.009}$	$0.211_{0.003}$	$20.6_{31.0}$
	800	$0.739_{0.007}$	$0.261_{0.003}$	$7.7_{11.1}$
15	298.15	$0.982_{0.007}$	$0.0039_{0.0005}$	$14087.9_{1626.9}$
	400	$0.976_{0.013}$	$0.0227_{0.0009}$	$848.5_{354.2}$
	500	$0.920_{0.036}$	$0.080_{0.003}$	$198.2_{145.0}$
	600	$0.850_{0.019}$	$0.150_{0.003}$	$69.3_{66.6}$
	700	$0.790_{0.007}$	$0.210_{0.002}$	$19.6_{9.5}$
	800	$0.739_{0.005}$	$0.261_{0.003}$	$18.8_{9.9}$
20	298.15	$0.981_{0.006}$	$0.0038_{0.0002}$	$14991.3_{3460.0}$
	400	$0.975_{0.007}$	$0.0238_{0.0008}$	$1180.3_{216.5}$
	500	$0.920_{0.013}$	$0.080_{0.001}$	$198.9_{52.4}$
	600	$0.854_{0.015}$	$0.146_{0.004}$	$95.4_{102.9}$

	700	$0.789_{0.004}$	$0.211_{0.002}$	$23.8_{17.9}$
	800	$0.738_{0.008}$	$0.262_{0.003}$	$23.8_{16.1}$
25	298.15	$0.980_{0.007}$	$0.0042_{0.0002}$	$16296.5_{2435.5}$
	400	$0.975_{0.007}$	$0.0237_{0.0005}$	$1399.9_{250.4}$
	500	$0.919_{0.013}$	$0.081_{0.003}$	$317.0_{64.1}$
	600	$0.851_{0.012}$	$0.149_{0.002}$	$66.5_{14.9}$
	700	$0.790_{0.017}$	$0.210_{0.003}$	$41.9_{16.1}$
	800	$0.739_{0.007}$	$0.261_{0.002}$	$22.3_{7.2}$
30	298.15	$0.977_{0.004}$	$0.0045_{0.0001}$	$18294.1_{1193.7}$
	400	$0.974_{0.007}$	$0.0240_{0.0004}$	$1835.4_{280.6}$
	500	$0.920_{0.009}$	$0.080_{0.002}$	$321.2_{152.2}$
	600	$0.851_{0.007}$	$0.149_{0.001}$	$93.8_{38.5}$
	700	$0.788_{0.004}$	$0.212_{0.002}$	$38.8_{10.8}$
	800	$0.737_{0.005}$	$0.263_{0.002}$	$26.6_{9.2}$
40	298.15	$0.968_{0.018}$	$0.004_{0.001}$	$28068.9_{6672.2}$
	350	$0.982_{0.003}$	$0.0123_{0.0005}$	$6143.9_{687.0}$
	400	$0.973_{0.003}$	$0.0252_{0.0008}$	$2093.9_{335.0}$
	500	$0.921_{0.009}$	$0.0789_{0.0009}$	$416.3_{82.2}$
	600	$0.850_{0.006}$	$0.149_{0.001}$	$137.3_{40.6}$
	700	$0.789_{0.009}$	$0.211_{0.002}$	$64.5_{27.8}$
	800	$0.738_{0.004}$	$0.262_{0.001}$	$33.6_{12.3}$
50	298.15	$0.963_{0.009}$	$0.0053_{0.0002}$	$31646.6_{3049.3}$
	350	$0.980_{0.005}$	$0.0140_{0.0009}$	$5933.9_{326.7}$
	400	$0.971_{0.011}$	$0.0262_{0.0004}$	$2366.1_{319.5}$
	500	$0.920_{0.008}$	$0.0798_{0.0008}$	$521.2_{129.5}$
	600	$0.851_{0.009}$	$0.149_{0.002}$	$166.9_{25.5}$
	700	$0.788_{0.005}$	$0.212_{0.002}$	$76.1_{13.3}$
	800	$0.737_{0.006}$	$0.263_{0.002}$	$46.9_{9.0}$
60	298.15	$0.966_{0.003}$	$0.0057_{0.0004}$	$28489.5_{2156.5}$
	350	$0.978_{0.003}$	$0.0142_{0.0002}$	$7797.5_{635.3}$
	400	$0.970_{0.006}$	$0.0275_{0.0005}$	$2823.2_{218.8}$
	500	$0.920_{0.007}$	$0.079_{0.001}$	$601.1_{59.2}$
	600	$0.850_{0.008}$	$0.149_{0.002}$	$214.7_{42.3}$
	700	$0.787_{0.007}$	$0.213_{0.002}$	$77.7_{9.5}$
	800	$0.737_{0.004}$	$0.263_{0.001}$	$61.3_{16.9}$

Table S7: Mole fractions of CO_2, H_2, and HCOOH obtained using Monte Carlo simulations in the grand-canonical ensemble in the Fe-MOF-74 framework at 298.15 - 800 K and 1 - 60 bar. The mole fractions of CO_2, H_2, and HCOOH at reaction equilibrium obtained from the Continuous Fractional Component Monte Carlo simulations in the Reaction Ensemble[9] were used in the GCMC simulations as input. The subscripts show uncertainties computed using error propagation rules.

P/[bar]	T/[K]	x_{CO_2}	x_{H_2}	x_{HCOOH} / 10^{-6}
1	298.15	$0.996_{0.012}$	$0.0023_{0.0003}$	$1595.0_{308.3}$
	400	$0.972_{0.027}$	$0.028_{0.004}$	$17.3_{35.5}$
	500	$0.913_{0.032}$	$0.087_{0.003}$	$0.9_{1.7}$
	600	$0.844_{0.011}$	$0.156_{0.005}$	0_{0}
	700	$0.782_{0.033}$	$0.218_{0.010}$	0_{0}
	800	$0.729_{0.025}$	$0.271_{0.009}$	0_{0}
5	298.15	$0.991_{0.008}$	$0.0035_{0.0004}$	$5511.9_{677.4}$
	400	$0.973_{0.019}$	$0.0262_{0.0008}$	$346.4_{138.6}$
	500	$0.913_{0.028}$	$0.087_{0.005}$	$41.3_{67.0}$
	600	$0.845_{0.008}$	$0.155_{0.002}$	$16.2_{22.4}$
	700	$0.784_{0.013}$	$0.216_{0.006}$	$18.3_{40.4}$
	800	$0.730_{0.004}$	$0.270_{0.004}$	0_{0}
10	298.15	$0.989_{0.007}$	$0.0039_{0.0006}$	$7503.4_{1927.5}$
	400	$0.974_{0.027}$	$0.0258_{0.0009}$	$662.6_{264.1}$
	500	$0.914_{0.021}$	$0.086_{0.002}$	$156.3_{112.6}$
	600	$0.843_{0.016}$	$0.157_{0.003}$	$34.8_{18.2}$
	700	$0.782_{0.010}$	$0.218_{0.003}$	$18.6_{7.2}$
	800	$0.731_{0.013}$	$0.269_{0.004}$	$4.0_{7.7}$
15	298.15	$0.984_{0.008}$	$0.0038_{0.0003}$	$12339.0_{1417.9}$
	400	$0.974_{0.015}$	$0.0252_{0.0007}$	$934.2_{222.5}$
	500	$0.914_{0.031}$	$0.085_{0.003}$	$186.5_{40.0}$
	600	$0.845_{0.018}$	$0.155_{0.003}$	$39.1_{17.5}$
	700	$0.781_{0.008}$	$0.219_{0.002}$	$19.2_{19.7}$
	800	$0.730_{0.003}$	$0.270_{0.002}$	$12.0_{17.6}$
20	298.15	$0.983_{0.004}$	$0.0042_{0.0004}$	$12733.8_{1301.1}$
	400	$0.973_{0.004}$	$0.0260_{0.0005}$	$1190.3_{86.2}$
	500	$0.915_{0.005}$	$0.085_{0.001}$	$200.5_{58.7}$
	600	$0.844_{0.009}$	$0.156_{0.002}$	$63.7_{29.2}$

	700	0.782 $_{0.005}$	0.217 $_{0.002}$	35.5 $_{24.1}$
	800	0.731 $_{0.004}$	0.269 $_{0.004}$	13.7 $_{6.4}$
25	298.15	0.981 $_{0.005}$	0.0045 $_{0.0004}$	14375.7 $_{3028.5}$
	400	0.973 $_{0.006}$	0.0258 $_{0.0005}$	1180.3 $_{147.2}$
	500	0.915 $_{0.011}$	0.085 $_{0.001}$	229.4 $_{36.0}$
	600	0.845 $_{0.009}$	0.155 $_{0.002}$	58.9 $_{9.5}$
	700	0.781 $_{0.008}$	0.219 $_{0.002}$	34.9 $_{14.4}$
	800	0.731 $_{0.007}$	0.269 $_{0.002}$	24.3 $_{10.7}$
30	298.15	0.981 $_{0.007}$	0.0048 $_{0.0005}$	14334.3 $_{1627.2}$
	400	0.972 $_{0.004}$	0.0267 $_{0.0006}$	1658.1 $_{507.9}$
	500	0.915 $_{0.018}$	0.084 $_{0.002}$	263.8 $_{98.5}$
	600	0.844 $_{0.012}$	0.156 $_{0.002}$	98.4 $_{27.7}$
	700	0.782 $_{0.005}$	0.218 $_{0.002}$	44.9 $_{9.8}$
	800	0.731 $_{0.006}$	0.269 $_{0.002}$	25.4 $_{17.7}$
40	298.15	0.976 $_{0.006}$	0.0054 $_{0.0003}$	18536.2 $_{3392.9}$
	350	0.981 $_{0.003}$	0.0135 $_{0.0005}$	5500.7 $_{642.9}$
	400	0.971 $_{0.004}$	0.0273 $_{0.0006}$	1877.4 $_{174.8}$
	500	0.914 $_{0.015}$	0.085 $_{0.001}$	425.3 $_{131.0}$
	600	0.844 $_{0.008}$	0.155 $_{0.002}$	129.3 $_{49.8}$
	700	0.780 $_{0.011}$	0.220 $_{0.003}$	51.8 $_{14.7}$
	800	0.731 $_{0.007}$	0.269 $_{0.002}$	37.8 $_{9.5}$
50	298.15	0.972 $_{0.005}$	0.0058 $_{0.0002}$	22638.2 $_{3577.5}$
	350	0.979 $_{0.005}$	0.0146 $_{0.0003}$	6342.0 $_{460.8}$
	400	0.969 $_{0.005}$	0.0284 $_{0.0003}$	2126.2 $_{95.5}$
	500	0.915 $_{0.010}$	0.084 $_{0.002}$	496.2 $_{131.3}$
	600	0.843 $_{0.006}$	0.157 $_{0.002}$	159.7 $_{42.1}$
	700	0.781 $_{0.004}$	0.219 $_{0.003}$	70.2 $_{9.4}$
	800	0.730 $_{0.006}$	0.270 $_{0.002}$	47.8 $_{18.9}$
60	298.15	0.971 $_{0.005}$	0.0067 $_{0.0006}$	22258.6 $_{2272.4}$
	350	0.978 $_{0.004}$	0.0153 $_{0.0004}$	6373.0 $_{225.8}$
	400	0.968 $_{0.004}$	0.0295 $_{0.0006}$	2668.8 $_{338.2}$
	500	0.916 $_{0.010}$	0.084 $_{0.001}$	495.6 $_{55.1}$
	600	0.843 $_{0.004}$	0.157 $_{0.001}$	174.1 $_{28.2}$
	700	0.781 $_{0.005}$	0.219 $_{0.002}$	89.6 $_{13.5}$
	800	0.729 $_{0.002}$	0.271 $_{0.001}$	62.1 $_{8.9}$

Table S8: Mole fractions of CO_2, H_2, and HCOOH obtained using Monte Carlo simulations in the grand-canonical ensemble in the Mn-MOF-74 framework at 298.15 - 800 K and 1 - 60 bar. The mole fractions of CO_2, H_2, and HCOOH at reaction equilibrium obtained from the Continuous Fractional Component Monte Carlo simulations in the Reaction Ensemble[9] were used in the GCMC simulations as input. The subscripts show uncertainties computed using error propagation rules.

P/[bar]	T/[K]	x_{CO_2}	x_{H_2}	x_{HCOOH} / 10^{-6}
1	298.15	$0.997_{0.015}$	$0.0025_{0.0004}$	$420.7_{179.3}$
	400	$0.968_{0.021}$	$0.032_{0.004}$	$17.8_{46.2}$
	500	$0.910_{0.041}$	$0.090_{0.008}$	$5.4_{11.7}$
	600	$0.843_{0.022}$	$0.157_{0.004}$	0_{0}
	700	$0.781_{0.033}$	$0.219_{0.010}$	0_{0}
	800	$0.729_{0.006}$	$0.271_{0.009}$	0_{0}
5	298.15	$0.995_{0.004}$	$0.0035_{0.0001}$	$1396.2_{251.0}$
	400	$0.972_{0.026}$	$0.028_{0.004}$	$58.2_{74.0}$
	500	$0.909_{0.015}$	$0.091_{0.002}$	$13.3_{10.1}$
	600	$0.841_{0.009}$	$0.159_{0.002}$	$13.5_{14.9}$
	700	$0.780_{0.015}$	$0.220_{0.005}$	$10.6_{26.7}$
	800	$0.731_{0.010}$	$0.269_{0.003}$	$8.7_{20.1}$
10	298.15	$0.994_{0.009}$	$0.0037_{0.0004}$	$1823.0_{605.6}$
	400	$0.972_{0.013}$	$0.0281_{0.0009}$	$220.4_{114.7}$
	500	$0.911_{0.015}$	$0.089_{0.002}$	$48.0_{31.6}$
	600	$0.841_{0.013}$	$0.158_{0.002}$	$19.7_{13.1}$
	700	$0.780_{0.005}$	$0.220_{0.002}$	$11.5_{13.1}$
	800	$0.733_{0.010}$	$0.267_{0.003}$	$8.8_{17.4}$
15	298.15	$0.993_{0.008}$	$0.0036_{0.0006}$	$3488.4_{458.9}$
	400	$0.972_{0.014}$	$0.028_{0.001}$	$393.1_{87.4}$
	500	$0.910_{0.012}$	$0.090_{0.001}$	$52.2_{29.3}$
	600	$0.841_{0.009}$	$0.159_{0.002}$	$21.5_{10.6}$
	700	$0.781_{0.011}$	$0.219_{0.003}$	$19.2_{23.4}$
	800	$0.729_{0.005}$	$0.271_{0.002}$	$14.7_{7.5}$
20	298.15	$0.993_{0.006}$	$0.0044_{0.0006}$	$2739.3_{1152.4}$
	400	$0.972_{0.010}$	$0.0276_{0.0004}$	$493.1_{70.9}$
	500	$0.911_{0.009}$	$0.089_{0.002}$	$91.5_{30.9}$
	600	$0.841_{0.005}$	$0.159_{0.002}$	$46.3_{25.3}$

	700	0.779 0.005	0.221 0.002	17.8 5.8
	800	0.731 0.004	0.269 0.001	14.6 16.1
25	298.15	0.992 0.006	0.0043 0.0005	3931.5 1070.0
	400	0.971 0.017	0.029 0.002	511.4 256.4
	500	0.911 0.008	0.089 0.001	170.2 77.4
	600	0.841 0.008	0.159 0.001	58.0 13.6
	700	0.779 0.005	0.221 0.002	37.2 12.0
	800	0.730 0.005	0.270 0.002	19.0 8.6
30	298.15	0.992 0.005	0.0046 0.0004	3313.9 417.0
	400	0.972 0.013	0.027 0.002	511.7 194.3
	500	0.912 0.009	0.088 0.002	150.7 64.1
	600	0.840 0.009	0.160 0.002	54.5 17.5
	700	0.779 0.006	0.220 0.001	28.5 5.5
	800	0.729 0.006	0.271 0.003	30.0 8.6
40	298.15	0.990 0.006	0.0051 0.0004	5294.1 522.2
	350	0.985 0.004	0.0136 0.0005	1728.2 278.0
	400	0.970 0.008	0.0289 0.0004	874.0 169.2
	500	0.911 0.014	0.089 0.001	193.3 40.7
	600	0.841 0.009	0.159 0.002	77.6 21.8
	700	0.778 0.006	0.222 0.001	46.8 15.4
	800	0.730 0.004	0.270 0.003	26.3 19.0
50	298.15	0.988 0.007	0.0050 0.0004	6761.3 1764.2
	350	0.984 0.004	0.0145 0.0008	1951.9 311.5
	400	0.970 0.005	0.0295 0.0004	910.8 130.5
	500	0.912 0.003	0.0879 0.0008	257.6 44.9
	600	0.840 0.008	0.160 0.001	90.0 18.9
	700	0.779 0.008	0.221 0.002	57.1 11.7
	800	0.730 0.004	0.270 0.002	37.2 13.1
60	298.15	0.987 0.003	0.0064 0.0003	6127.1 587.4
	350	0.982 0.003	0.0156 0.0005	2367.8 104.6
	400	0.969 0.004	0.0305 0.0007	961.0 151.8
	500	0.911 0.005	0.0886 0.0004	286.7 39.5
	600	0.840 0.005	0.160 0.002	110.0 18.4
	700	0.778 0.005	0.222 0.002	69.7 20.3
	800	0.729 0.004	0.271 0.002	43.0 9.8

Table S9: Mole fractions of CO_2, H_2, and HCOOH obtained using Monte Carlo simulations in the grand-canonical ensemble in the Zn-MOF-74 framework at 298.15 - 800 K and 1 - 60 bar. The mole fractions of CO_2, H_2, and HCOOH at reaction equilibrium obtained from the Continuous Fractional Component Monte Carlo simulations in the Reaction Ensemble[9] were used in the GCMC simulations as input. The subscripts show uncertainties computed using error propagation rules.

P/[bar]	T/[K]	x_{CO_2}	x_{H_2}	x_{HCOOH} / 10^{-6}
1	298.15	$0.997_{0.023}$	$0.0031_{0.0004}$	$77.9_{70.1}$
	400	$0.969_{0.054}$	$0.031_{0.004}$	$0.0_{0.0}$
	500	$0.912_{0.030}$	$0.088_{0.003}$	$0.1_{0.3}$
	600	$0.843_{0.020}$	$0.157_{0.007}$	0_{0}
	700	$0.786_{0.015}$	$0.214_{0.007}$	0_{0}
	800	$0.735_{0.010}$	$0.265_{0.008}$	0_{0}
5	298.15	$0.996_{0.003}$	$0.0036_{0.0002}$	$289.3_{35.6}$
	400	$0.970_{0.021}$	$0.030_{0.001}$	$75.7_{65.0}$
	500	$0.911_{0.018}$	$0.089_{0.003}$	$16.8_{16.4}$
	600	$0.846_{0.005}$	$0.154_{0.001}$	$4.2_{4.8}$
	700	$0.787_{0.007}$	$0.213_{0.002}$	$7.8_{9.2}$
	800	$0.737_{0.009}$	$0.263_{0.003}$	0_{0}
10	298.15	$0.996_{0.002}$	$0.0036_{0.0003}$	$349.9_{100.7}$
	400	$0.971_{0.004}$	$0.0291_{0.0007}$	$92.6_{60.4}$
	500	$0.913_{0.006}$	$0.087_{0.001}$	$21.4_{12.8}$
	600	$0.847_{0.017}$	$0.153_{0.003}$	$15.6_{22.4}$
	700	$0.786_{0.005}$	$0.214_{0.002}$	$7.6_{2.9}$
	800	$0.738_{0.007}$	$0.262_{0.002}$	$4.2_{5.5}$
15	298.15	$0.995_{0.005}$	$0.0041_{0.0004}$	$702.7_{274.3}$
	400	$0.971_{0.011}$	$0.0284_{0.0007}$	$102.3_{20.3}$
	500	$0.912_{0.011}$	$0.088_{0.001}$	$39.5_{32.3}$
	600	$0.845_{0.010}$	$0.155_{0.002}$	$19.2_{16.5}$
	700	$0.786_{0.006}$	$0.214_{0.002}$	$14.5_{12.2}$
	800	$0.737_{0.003}$	$0.263_{0.002}$	$9.9_{13.9}$
20	298.15	$0.995_{0.004}$	$0.0044_{0.0003}$	$783.8_{197.1}$
	400	$0.971_{0.009}$	$0.0284_{0.0008}$	$159.1_{29.9}$
	500	$0.912_{0.014}$	$0.088_{0.001}$	$44.0_{16.8}$
	600	$0.846_{0.005}$	$0.154_{0.002}$	$35.0_{26.8}$

	700	0.786 0.009	0.214 0.002	18.3 13.5
	800	0.737 0.005	0.263 0.002	10.4 11.9
25	298.15	0.994 0.004	0.0045 0.0001	990.3 159.0
	400	0.971 0.009	0.0283 0.0004	171.6 53.3
	500	0.912 0.013	0.087 0.001	77.8 35.3
	600	0.845 0.004	0.155 0.002	32.6 26.8
	700	0.786 0.008	0.214 0.002	20.3 12.9
	800	0.737 0.006	0.263 0.003	30.8 20.0
30	298.15	0.994 0.004	0.0048 0.0001	821.8 267.2
	400	0.971 0.007	0.029 0.001	253.4 34.1
	500	0.913 0.007	0.086 0.001	88.9 43.7
	600	0.846 0.007	0.154 0.001	38.1 20.7
	700	0.786 0.004	0.2136 0.0008	29.5 14.7
	800	0.738 0.006	0.262 0.002	14.8 12.5
40	298.15	0.993 0.003	0.0055 0.0004	1231.4 399.7
	350	0.985 0.003	0.0140 0.0009	549.5 95.2
	400	0.971 0.004	0.0291 0.0003	310.5 43.0
	500	0.914 0.009	0.0861 0.0007	90.4 9.5
	600	0.846 0.004	0.1544 0.0009	51.1 22.4
	700	0.785 0.004	0.215 0.001	34.4 21.2
	800	0.737 0.004	0.2633 0.0009	32.0 11.2
50	298.15	0.993 0.002	0.0058 0.0001	1474.1 222.6
	350	0.984 0.002	0.0149 0.0003	648.1 117.4
	400	0.970 0.004	0.030 0.001	347.0 96.4
	500	0.913 0.002	0.0867 0.0006	135.7 12.7
	600	0.845 0.005	0.1546 0.0008	71.9 12.2
	700	0.785 0.003	0.215 0.001	61.4 14.8
	800	0.736 0.003	0.264 0.001	30.9 11.2
60	298.15	0.992 0.006	0.0065 0.0004	1621.5 771.8
	350	0.984 0.007	0.0151 0.0008	736.2 309.8
	400	0.968 0.004	0.0315 0.0001	438.8 67.5
	500	0.913 0.013	0.087 0.001	157.3 54.0
	600	0.845 0.002	0.1549 0.0009	77.2 16.7
	700	0.784 0.007	0.216 0.002	57.0 12.1
	800	0.736 0.004	0.264 0.002	46.3 5.6

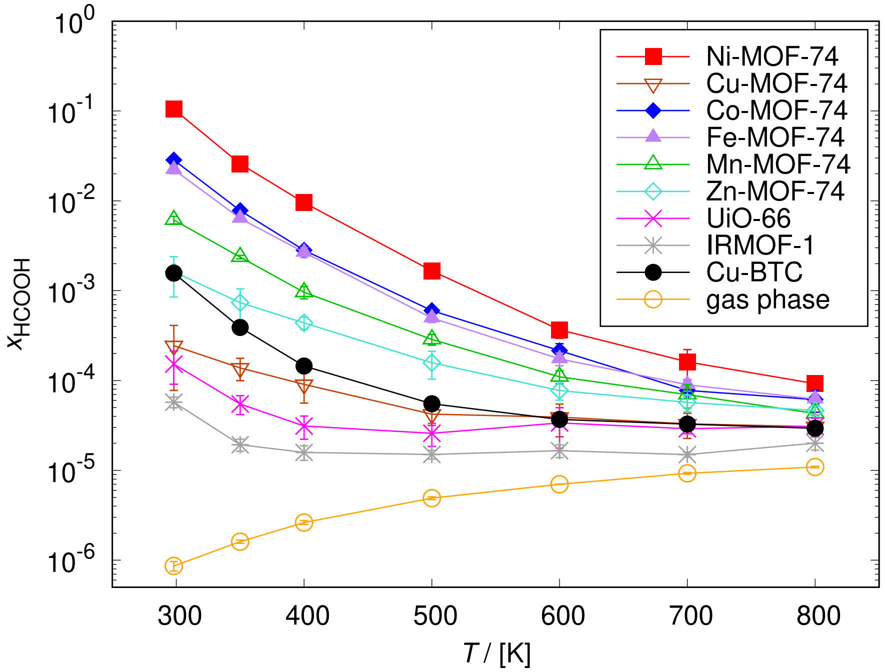

Figure S6. HCOOH mole fractions computed from Monte Carlo simulations in the grand-canonical ensemble in M-MOF-74 compared to the literature data for UiO-66, Cu-BTC, IRMOF-1, and the gas phase[9] at 298.15 - 800 K and 60 bar. The mole fractions of HCOOH computed from the Rx/CFC simulations in the study by Wasik et al.[9] are used as an input for the GCMC ensemble. The HCOOH mole fractions decrease with the increasing temperature. The highest HCOOH production resulted from the effect of confinement in Ni-MOF-74 at 298.15 K with a mole fraction equal to ca. 0.1.

(a)

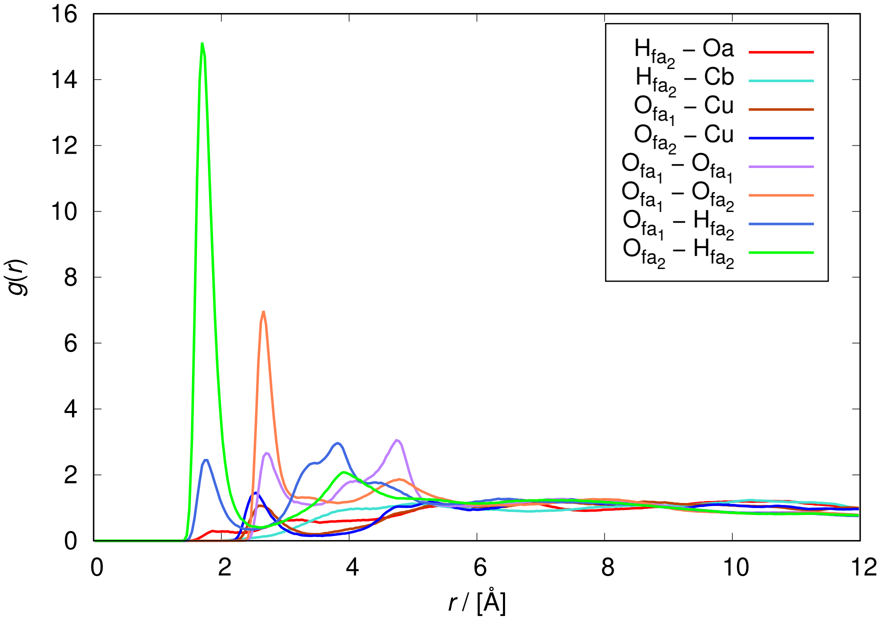

(b)

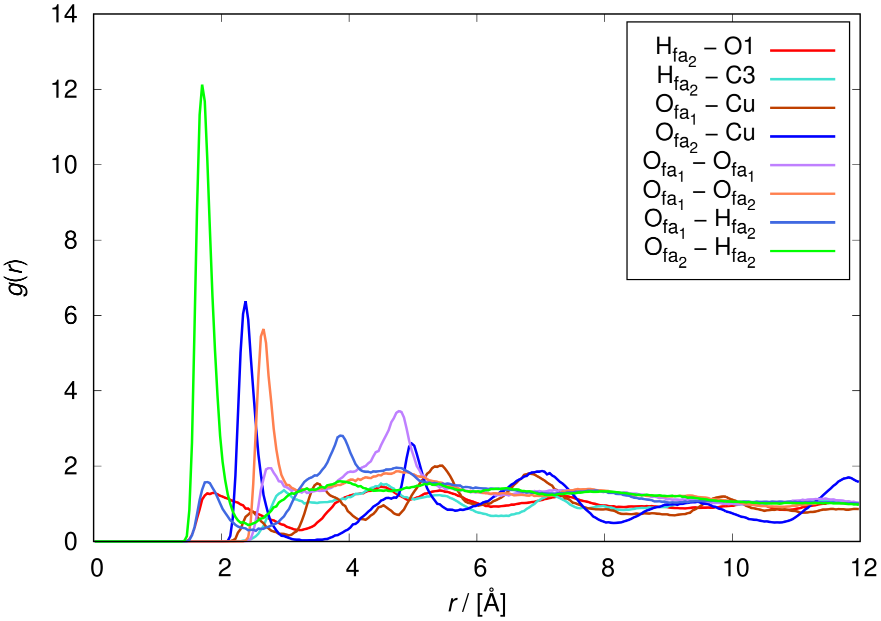

Figure S7. Radial distribution functions for 50 molecules of HCOOH in MOFs at 298 K: (a) Cu-MOF-74, and (b) Cu-BTC. While ca. 6 molecules are present in Cu-BTC within the preferential distance to the metal centers of 2.4 Å,[9] in Cu-MOF-74 only ca. 1.5 adsorbed molecules of HCOOH are within the distance of 2.54 Å to the metal centers. This difference in intensity is due to the higher charge on the metal center in Cu-BTC compared to Cu-MOF-74. The same effect is observed for the most electronegative ligand atom of the frameworks. The stronger electronegativity of the oxygen atom in Cu-BTC leads to a higher intensity of HCOOH adsorption oriented towards this atom (H_{fa2}-O1) that in Cu-MOF-74 (H_{fa2}-Oa). The formation of hydrogen bonds between HCOOH molecules is slightly more intense in Cu-MOF-74 than in Cu-BTC due to weaker interactions with the framework. The simulations were performed using the RASPA software package.[17,18]

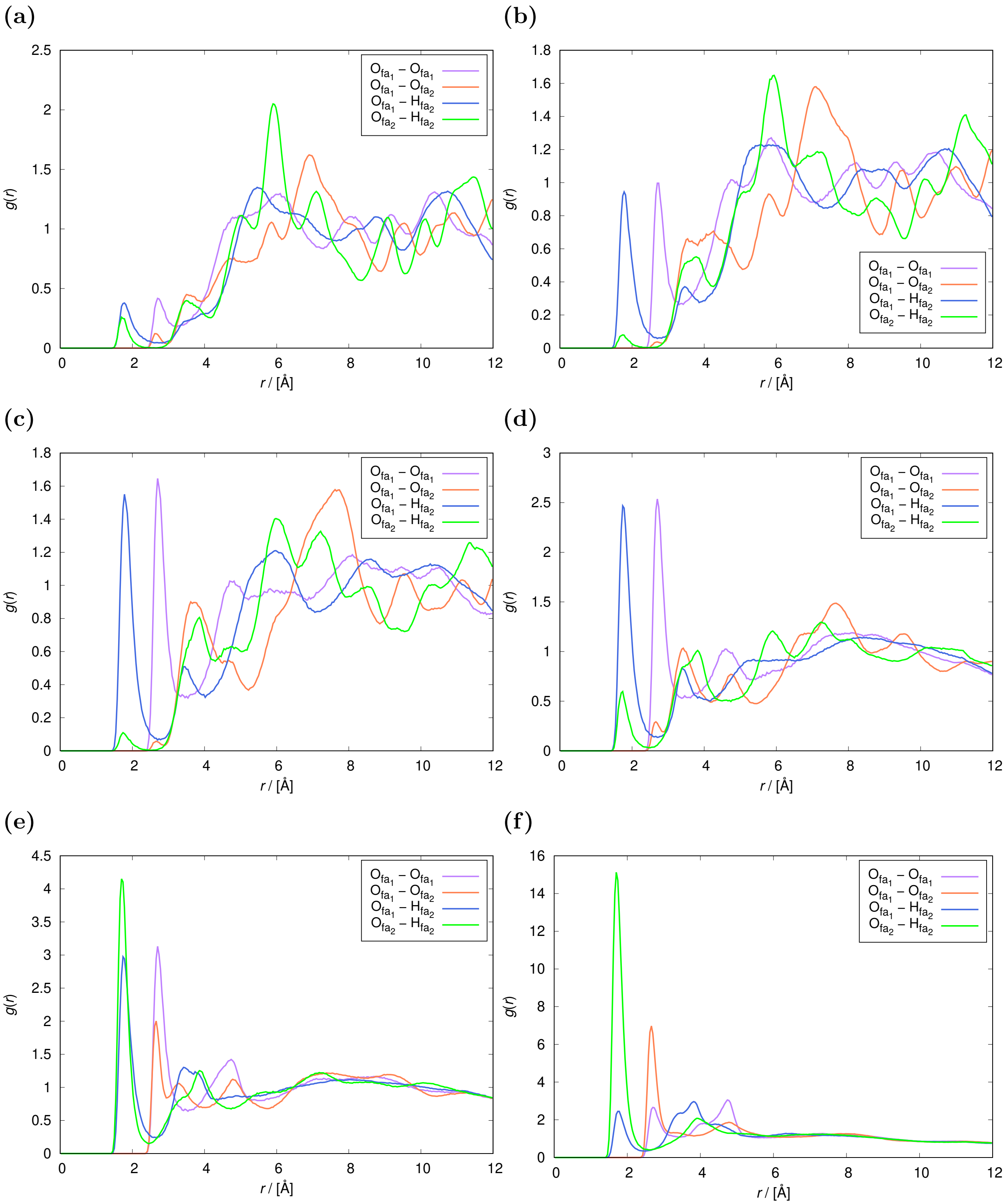

Figure S8. Radial distribution functions simulated for 50 molecules of HCOOH at 298 K in: (a) Ni-MOF-74, (b) Co-MOF-74, (c) Fe-MOF-74, (d) Mn-MOF-74, (e) Zn-MOF-74, and (f) Cu-MOF-74. The intensity of hydrogen bond formation increase with decreasing affinity of HCOOH with the framework. In Zn-, and Cu-MOF-74, the HCOOH dimerization is found to be prevalent over the hydrogen bonds-driven nucleation. The simulations were performed using the RASPA software package.[17,18]

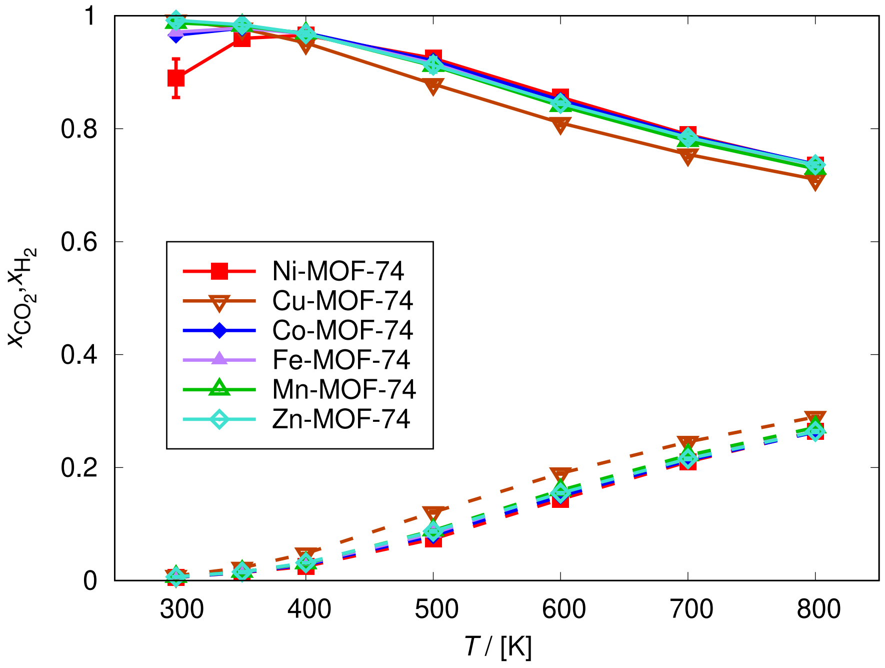

Figure S9. CO_2 and H_2 mole fractions computed from Monte Carlo simulations in the grand-canonical ensemble in M-MOF-74. The mole fractions computed from the Rx/CFC simulations are used as an input for the GCMC ensemble. The simulations were carried out at 298.15 - 800 K and 60 bar. The data points of CO_2 mole fractions are connected by the solid lines and the data points of H_2 mole fractions by the dashed lines to guide the eye. The mole fractions of CO_2 decrease with increasing temperature, except for Ni-, Co-, and Fe-MOF-74 at the temperature range 298.15 - 350 K, where the mole fraction of CO_2 slightly increases. The increase is caused by the large decrease in HCOOH production, affecting mole fractions of the other components. The number of adsorbed CO_2 molecules decreases throughout the range of studied temperatures. The mole fractions of H_2 increase with temperature.

References

(1) Harris, J. G.; Yung, K. H. Carbon Dioxide's Liquid-Vapor Coexistence Curve And Critical Properties as Predicted by a Simple Molecular Model. *Journal of Physical Chemistry* **1995**, *99*, 12021–12024.

(2) García-Sánchez, A.; Ania, C. O.; Parra, J. B.; Dubbeldam, D.; Vlugt, T. J. H.; Krishna, R.; Calero, S. Transferable Force Field for Carbon Dioxide Adsorption in Zeolites. *Journal of Physical Chemistry C* **2009**, *113*, 8814–8820.

(3) Darkrim, F.; Levesque, D. Monte Carlo Simulations of Hydrogen Adsorption in Single-Walled Carbon Nanotubes. *Journal of Chemical Physics* **1998**, *109*, 4981–4984.

(4) Salas, F. J.; Nunez-Rojas, E.; Alejandre, J. Stability of Formic Acid/Pyridine and Isonicotinamide/Formamide Cocrystals by Molecular Dynamics Simulations. *Theoretical Chemistry Accounts* **2017**, *136*, 1–12.

(5) Allen, M. P.; Tildesley, D. J. *Computer Simulation of Liquids*, 2nd ed.; Oxford University Press, 2017.

(6) Wasik, D. O.; Vicent-Luna, J. M.; Luna-Triguero, A.; Dubbeldam, D.; Vlugt, T. J. H.; Calero, S. The impact of metal centers in the M-MOF-74 series on carbon dioxide and hydrogen separation. *Separation and Purification Technology* **2024**, *339*, 126539.

(7) Queen, W. L.; Hudson, M. R.; Bloch, E. D.; Mason, J. A.; Gonzalez, M. I.; Lee, J. S.; Gygi, D.; Howe, J. D.; Lee, K.; Darwish, T. A.; others Comprehensive Study of Carbon Dioxide Adsorption in the Metal–Organic Frameworks M_2 (dobdc)(M= Mg, Mn, Fe, Co, Ni, Cu, Zn). *Chemical Science* **2014**, *5*, 4569–4581.

(8) Yu, D.; Yazaydin, A. O.; Lane, J. R.; Dietzel, P. D. C.; Snurr, R. Q. A Combined Experimental and Quantum Chemical Study of CO_2 Adsorption in the Metal–Organic Framework CPO-27 with Different Metals. *Chemical Science* **2013**, *4*, 3544–3556.

(9) Wasik, D. O.; Martín-Calvo, A.; Gutiérrez-Sevillano, J. J.; Dubbeldam, D.; Vlugt, T. J. H.; Calero, S. Enhancement of Formic Acid Production from Carbon Dioxide Hydrogenation Using Metal-Organic Frameworks: Monte Carlo Simulation Study. *Chemical Engineering Journal* **2023**, *467*, 143432.

(10) Shi, W.; Maginn, E. J. Continuous Fractional Component Monte Carlo: An Adaptive Biasing Method for Open System Atomistic Simulations. *Journal of Chemical Theory and Computation* **2007**, *3*, 1451–1463.

(11) Shi, W.; Maginn, E. J. Improvement in Molecule Exchange Efficiency in Gibbs Ensemble Monte Carlo: Development and Implementation of the Continuous Fractional Component Move. *Journal of Computational Chemistry* **2008**, *29*, 2520–2530.

(12) Rahbari, A.; Hens, R.; Ramdin, M.; Moultos, O. A.; Dubbeldam, D.; Vlugt, T. J. H. Recent Advances in the Continuous Fractional Component Monte Carlo Methodology. *Molecular Simulation* **2021**, *47*, 804–823.

(13) Smith, W. R.; Triska, B. The Reaction Ensemble Method for the Computer Simulation of Chemical and Phase Equilibria. I. Theory and Basic Examples. *Journal of Chemical Physics* **1994**, *100*, 3019–3027.

(14) Johnson, J. K.; Panagiotopoulos, A. Z.; Gubbins, K. E. Reactive Canonical Monte Carlo. *Molecular Physics* **1994**, *81*, 717–733.

(15) Poursaeidesfahani, A.; Hens, R.; Rahbari, A.; Ramdin, M.; Dubbeldam, D.; Vlugt, T. J. H. Efficient Application of Continuous Fractional Component Monte Carlo in the Reaction Ensemble. *Journal of Chemical Theory and Computation* **2017**, *13*, 4452–4466.

(16) Luna-Triguero, A.; Vicent-Luna, J. M.; Madero-Castro, R. M.; Gómez-Álvarez, P.; Calero, S. Acetylene Storage and Separation Using Metal–Organic Frameworks with Open Metal Sites. *ACS Applied Materials & Interfaces* **2019**, *11*, 31499–31507.

(17) Dubbeldam, D.; Calero, S.; Ellis, D. E.; Snurr, R. Q. RASPA: Molecular Simulation Software for Adsorption and Diffusion in Flexible Nanoporous Materials. *Molecular Simulation* **2016**, *42*, 81–101.

(18) Dubbeldam, D.; Torres-Knoop, A.; Walton, K. S. On the Inner Workings of Monte Carlo Codes. *Molecular Simulation* **2013**, *39*, 1253–1292.
